# Grafting of Diazonium Salts on Surfaces: Application to Biosensors

**DOI:** 10.3390/bios10010004

**Published:** 2020-01-15

**Authors:** Dardan Hetemi, Vincent Noël, Jean Pinson

**Affiliations:** 1 Pharmacy Department, Medical Faculty, University of Prishtina, “Hasan Prishtina”, Rr. “Dëshmorët e Kombit” p.n., 10000 Prishtina, Kosovo; dardan.hetemi@uni-pr.edu; 2 Université de Paris, ITODYS, CNRS, UMR 7086, 15 rue J-A de Baïf, F-75013 Paris, France; vincent.noel@univ-paris-diderot.fr

**Keywords:** diazonium, biosensor, surface modification

## Abstract

This review is divided into two parts; the first one summarizes the main features of surface modification by diazonium salts with a focus on most recent advances, while the second part deals with diazonium-based biosensors including small molecules of biological interest, proteins, and nucleic acids.

## 1. An Overview of the Reactions of Diazonium Salts with Surfaces

### 1.1. The Principle of the Reaction

**Scheme 1:**
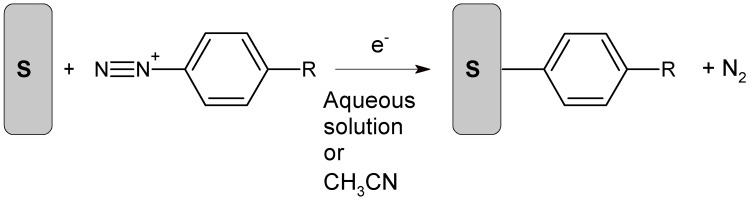
Electrografting of diazonium salts.

The simplest way to represent the grafting of aryldiazonium salts on surfaces is shown in Scheme 1.

A substituted aryldiazonium salt dissolved in an aqueous medium or in acetonitrile (ACN) is reduced by one electron; as a result, the electrode is modified by aryl groups [1]. This is a very simple reaction with many experimental alternatives concerning the process itself, the surfaces, and the choice of the substituent. The result is a modified surface with strongly bonded aryl groups [2,3,4,5].

The main characteristics of this reaction are as follows:

* Diazonium salts are easily synthetized (isolated or not) from aromatic amines, many of which are commercially available.
* All surfaces can be modified by this reaction, conductive or not.
* The reaction can be performed by electrochemistry, spontaneously, by photochemistry, and by other methods.
* The resulting modification is very stable due to the formation of a covalent bond between the surface and the aryl group.
* The key species of this reaction is an aryl radical, and this reaction presents the typical behavior of radical reactions.
* The reaction provides most often disordered oligomers (“multilayers”).

Below, we review these different points with a focus on most recent examples.

### 1.2. Synthesis and Stability of Diazonium Salts

Surface modification of diazonium salts was achieved starting either from isolated salts or from solutions of in situ synthetized compounds [6]. The diazoniation was achieved with NaNO_2_ in acidic aqueous solution, or with *t*-butylnitrite or NOBF_4_ in ACN. As many aromatic amines are commercially available, the use of simple diazonium salts only necessitates a minimum effort of synthesis.

The stability of diazonium salts was measured; 4-nitrobenzenediazonium tosylate and tetrafluoroborate have half-life times close to 4.5 years, whereas 4-nitrobenzenediazonium triflate is much more stable with a half-life time of 46 years. These half-lives are more than sufficient to obtain reliable experiments [7]. However, chlorides and other salts are explosive.

When dissolved in ACN or aqueous acidic solution, the diazonium salts are present as Ar-N≡N^+^; however, as the pH increases in aqueous solution, they exist as Ar-N=N-OH (diazohydroxide) and Ar-N=N-O^−^ (diazoate). The first pKa of 4-nitrobenzenediazonium is 5.24. In ACN and aqueous acidic solution, diazonium salts are relatively stable (k_dec_ = 8.26 × 10^−6^ s^−1^ in aqueous acidic solution); however, as the pH increases, the rate of decomposition of 4-methylbenzenediazonium increases (k_dec_ ≈ 1.3 × 10^−2^ and 3.5 × 10^−2^ s^−1^ at pH 4 and 8, respectively (60 °C, in EtOH/H_2_O) [8,9]. The dediazoniation occurs either heterolytically or homolytically to give either a carbocation or a radical; as concerns the grafting reaction, there is only a very minor involvement of carbocations in the case of spontaneous reactions [10].

### 1.3. Different Grafting Methods of Diazonium Salts

As diazonium salts are easily reduced, a number of methods permit their homolytic dediazoniation; the most used ones are presented below.

**Figure 1:**
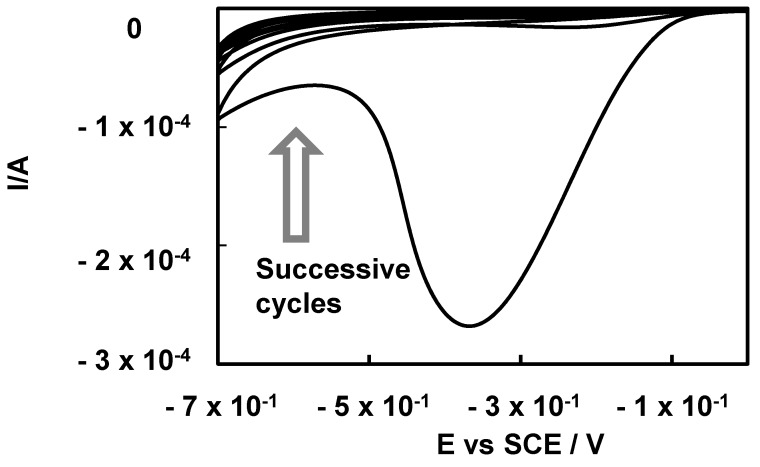
Cyclic voltammograms (scans 1–3) at fluorine tin oxide (FTO) electrode in HCl 0.5 M + 2 × 10^−3^ M 4-aminobenzoic acid + 2.2 × 10^−3^ M NaNO_2_. Scan rate of 0.1 V∙s^−1^. By courtesy of C. Cannizzo.

*By electrochemistry.* As indicated in Scheme 1, triggering of the reaction is possible by electrochemistry. The voltammograms obtained for the reduction of diazonium salts are very characteristic, showing a broad irreversible wave at potentials close to 0 V/SCE (Saturated Calomel Electrode). The wave is irreversible due to the cleavage and loss of N_2_; it is broad because the surface is modified during the voltammogram. Upon repetitive scanning, this wave decreases and finally disappears as an insulating organic film forms on the electrode (Figure 1) [11]. Repetitive cyclic voltammetry (5–10 cycles) and chronoamperometry at a potential equal or negative to the reduction peak are common methods for the modification of electrodes.

*By spontaneous reaction.* As the pH of an aqueous solution increases, the diazonium cation is transformed into the much more unstable diazohydroxide and diazoate. Consecutive homolytic cleavage of these species provides a radical that reacts with the surface. For example, a diazonium salt bearing an Iniferter initiator (a group acting as initiator, transfer, and terminator agent of controlled free radical polymerization) was grafted on isolating silica particles in basic medium to give a silica core@poly(acrylic acid) shell [12]. Trifluoromethylphenyl layers were grafted by immersing SnO_2_ plates in an aqueous solution of trifluoromethylbenzenediazonium for 8 h, in the dark [13]. 

*By reducing surfaces.* As diazonium salts are very easily reduced (Figure 1), materials such as copper, iron, and even carbon are reducing enough to perform the spontaneous grafting reaction onto their surface. A nanostructured and oxidized copper surface was grafted spontaneously by a perfluorobenzenediazonium salt [14] or an Iniferter initiator [15] through a one-pot reaction; the diazonium was prepared from the corresponding aniline in ACN + *t*-butylnitrite, and grafting took place spontaneously in the same solution. In the first case, a super-hydrophobic surface was obtained with a water contact angle of 158°, and, in the second one, it was possible to grow polyacrylic brushes by Iniferter polymerization.

Silica nanoparticles were modified with a molecule terminated by an aniline group that could be diazotized and grafted spontaneously on an iron surface at room temperature [16]. Anthraquinonediazonium prepared in ACN reacts with carbon black just by overnight reaction at room temperature [17], and graphene was modified with 3,5-bis difluorobenzenediazonium in acidic medium (the same medium used for the preparation of the diazonium cation) [18].

*By reducing reagents.* Even very mild reducing agents such as hypophosphorous acid, ascorbic acid, and iron powder are able to reduce diazonium cations leading to aryl radical formation. Coal powder was modified by 4-nitrophenyl groups via reduction of the corresponding diazonium cation by hypophosphorous acid. This modified coal embedded in reverse osmosis membranes improved their performance [19]. TiN is a ceramic material used in microelectronic devices, solar cells, electrical, interconnections, orthopedic prostheses, and cardiac valves. Its surface was modified by reaction of the mono-diazonium salt of *p*-phenylenediamine (NH_2_-C_6_H_4_-N≡N^+^) in the presence of hypophosphorous acid or iron powder [20]. This reaction offers the first step of an alternative to the metallization of titanium nitride by direct electrochemical deposition.

*By photochemistry.* Diazonium salts were grafted on metals gold, copper, and iron under UV (UltraViolet) light by irradiation in the presence of a photosensitizer (Ru(bipy)_3_^2+^ or eosin Y). In this way, gold and polyvinylchloride surfaces were modified by 4-phenylacetic, 4-carboxy, 4-methoxy, and 3,5-bis trifluoromethyl phenyl groups [21]. The reaction also took place under visible light by irradiation of charge transfer complexes such as 1,4-dimethoxybenzene and pentafluoro benzenediazonium cation (λ_max_ ~400 nm) [22].

**Figure 2:**
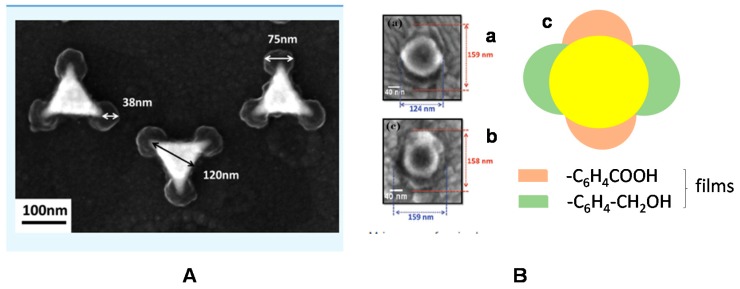
SEM image of (**A**) Au nanotriangles after irradiation with visible light in the presence of 4-[1-(2-bisthienyl)]benzenediazonium, (**B**) nanodiscs modified with visible light (Ba) with carboxyphenyl films grafted along the *Y*-direction, (Bb) additional hydroxyethyl phenyl films along the *X*-axis, (**B**c) Schematic presentation of the modified nanodisk. From References [23,24,25], with permission of the American Chemical Society (ACS) and the Royal Chemical Society, respectively.

Surface modification can also be triggered by localized surface plasmon excitation [23,24,25]. Using this approach, 4-[1-(2-bisthienyl)], 4-carboxy 4-hydroxyethyl benzenediazonium cations were grafted on gold nanostructures. Upon irradiation, hot collective oscillation of the conductive electrons at the particle surface provides the localized surface plasmon resonances (LSPR); hot electrons are generated on nanostructures particularly at the extremities of nanorods, nanotriangles, etc. These hot electrons induce the reduction of diazonium salts, the homolytic dediazoniation, and grafting at the extremities of gold nanostructures. In this way, regioselectively modified gold nanodiscs were obtained by successive use of two different light polarizations in the presence of two diazonium salts (Figure 2). Recently, diazonium-modified graphene-protected metal thin films (Cu) SPR biochips were designed for the detection of toxins [26].

### 1.4. The Different Surfaces That Can be Grafted

Many examples of grafting diazonium salts on glassy carbon, metals, and semiconductors are described [2]. Due to the current interest in nanoscience, more recent investigations examined one- and two-dimensional (1D and 2D) materials, as well as nanoparticles [27,28,29] (carbon nanotubes, graphene, graphene oxide, MoS_2_) as substrate.

*Carbon nanotubes (CNT).* A detailed investigation of the grafting of 4-iodobenzenediazonium on SWCNTs (single-walled carbon nanotubes) showed that (i) the bonded aryl groups were very stable, as they cleaved above 200 °C, (ii) a logarithmic correlation between the degree of functionalization and diazonium concentration was observed, and (iii) the maximum surface concentration was measured as one aryl group per 100 carbon atoms [30].

**Figure 3:**
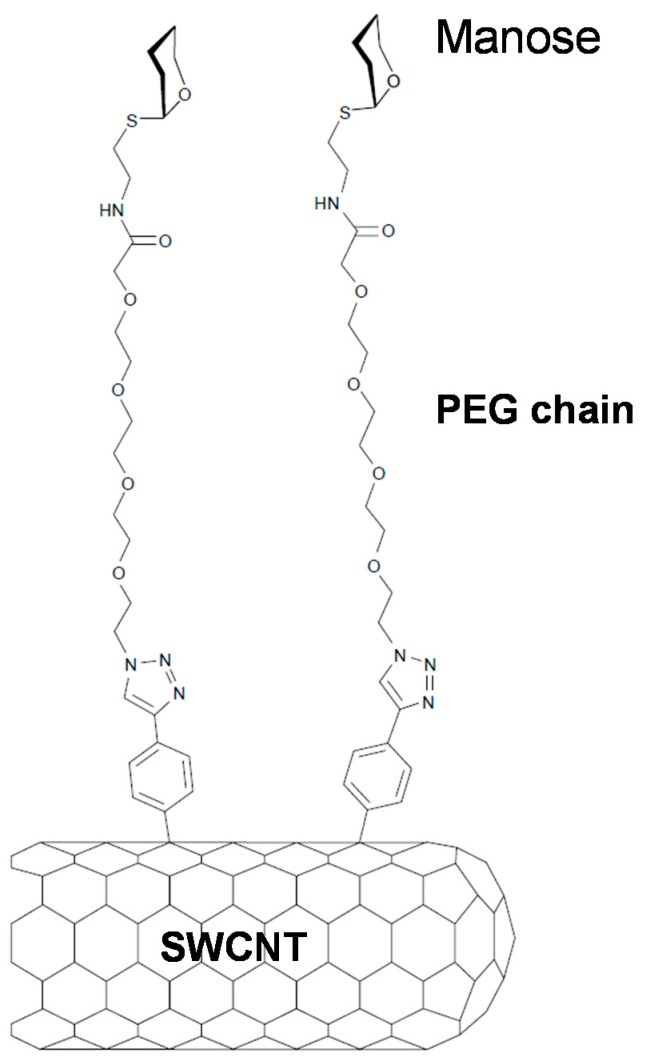
Single-walled carbon nanotube (SWCNT) modified with polyethyleneglycol (PEG) and mannose groups. From Reference [31] with permission of Elsevier.

In view of biological applications (antifouling properties), the surface of carbon nanotubes was modified in order to inhibit the growth of uropathogenic *Escherichia coli*. This was achieved by spontaneously grafting polyethyleneglycol (PEG) chains terminated by mannose at one and a benzenediazonium at the other end [31] (Figure 3).

**Figure 4:**
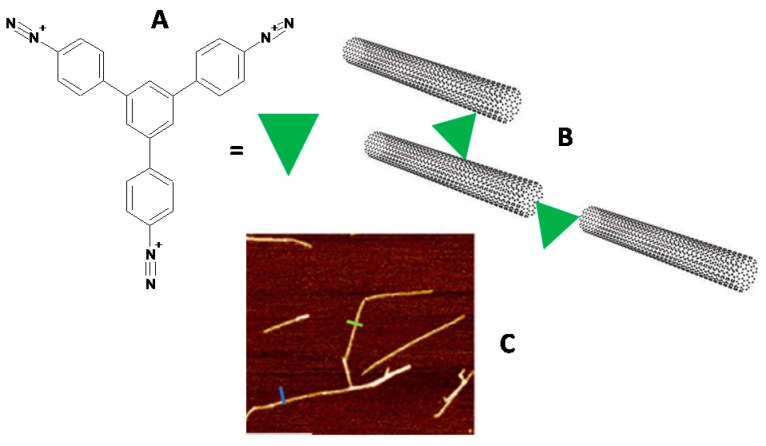
Arrays of SWCNTs bonded by reaction of diazonium salts.

Crosslinked assemblies of bonded CNTs were prepared using a molecule with three diazonium functional groups. The walls of the CNTs were firstly protected by wrapping DNA to limit their functionalization and favor the modification at nanotube end; then, the triple diazonium salt molecule was reacted spontaneously. The SWCNTs were bonded mainly through side-to-end junctions, and eventually through side-to-side interactions (Figure 4) [32].

*Graphene; graphene oxide (GO), reduced graphene oxide (RGO), highly oriented pyrolytic graphite (HOPG), carbon dots.* Graphene sheets were obtained by electrochemical exfoliation and functionalization of graphite using diazonium salts. Both functionalization and exfoliation occurred at the same time; in this way, mono- or few-layer graphene was functionalized and stabilized in situ before it aggregated; N_2_ generated during in situ diazonium reduction favored the separation of functionalized graphene sheets [33]. In planes or edges, grafting was easily achieved with diazonium salts; GO and RGO were modified with sulfophenyl groups up to 12 wt% [34].

The planes and edges of graphene (respectively, *sp*^2^ and *sp*^3^ carbons) have very different structures, but both react with diazonium salts under electrochemical conditions.

The surface modification of HOPG was examined at the micrometer scale [35] using scanning electrochemical cell microscopy where a dual-barrel micro-pipet explored the localized electrochemistry of the surface. In the presence of diazonium salts, grafting occurred along with the re-hybridization of surface carbons from *sp*^2^ to *sp*^3^ as confirmed by Raman spectroscopy; after diazonium modification, the D-band developed at ~1350 cm^−1^, diagnostic of the local *sp*^3^ carbons.

An electrode was constructed with only graphene edges exposed by cutting a connected graphene monolayer embedded in a polymer with an atomically sharp microtome knife. This graphene-edge electrode was modified by electrochemical reduction of 4-nitrobenzenediazonium. The cyclic voltammetry of 4-nitrophenyl films was observed, and the signal of the redox probe Fe(CN)_6_^3−/4−^ was completely inhibited, indicating a blocking of the electrode by the grafted film; this was confirmed by the Raman spectrum that indicated an increase of the D-band [36].

**Figure 5:**
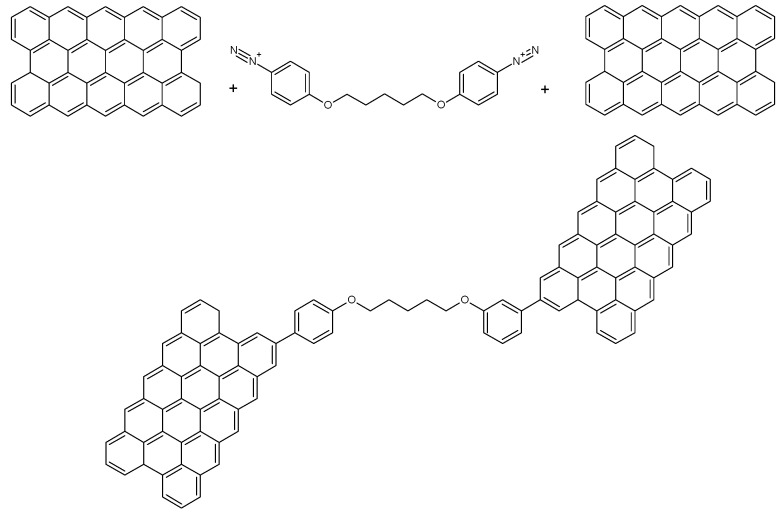
Binding graphene sheets using a bis-diazonium salt. From Reference [37] with permission of Wiley.

The reactivity of graphene edges was harnessed to prepare covalently bonded graphene nanoflakes further assembled by noncovalent interactions to give nanopapers. Binding of nanoflakes was achieved by use of a bis-diazonium salt. This diazonium cation was prepared in situ and grafted by increasing the temperature (Figure 5). Modification led to a 20% enhancement of the thermal conductivity, while the cross-plane thermal conductivity was boosted by 190% [37].

**Figure 6:**
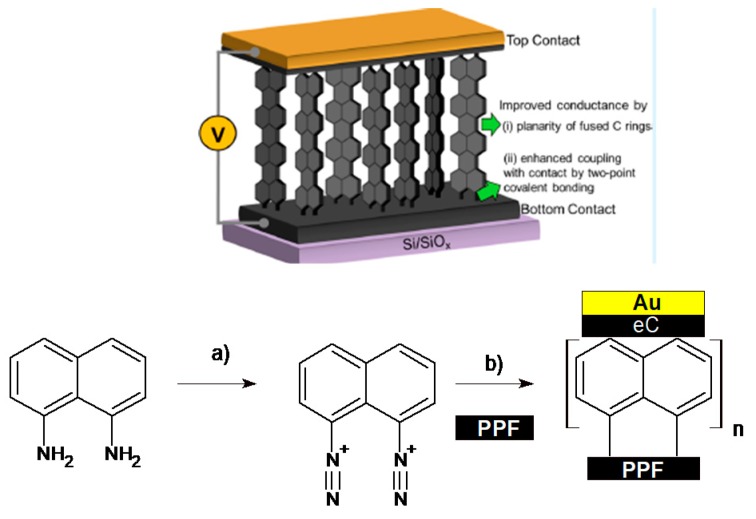
Molecular junction with carbon nanoribbons: (**a**) *t*-butylnitrite in ACN; (**b**) electrochemical reduction. PPF: pyrolyzed photoresist film, a very flat carbon surface, eC: electron-beam deposited carbon. From Reference [40] with permission of ACS.

It is also possible to prepare graphene ribbons from diazonium salts. Molecular junctions using aryl oligomers were obtained from diazonium salts [38]. An organic oligomeric aryl layer was grown on a conducting substrate and capped with, for example, a gold layer; this provided a molecular junction between two conducting materials [39]. Such molecular junctions are used in molecular electronic devices. These aryl oligomers were replaced by five-carbon-wide graphene ribbons (GR) with lengths of 2–12 nm; their conductance was more than one hundred times that observed for other molecular junctions of similar thicknesses. These nanoribbons were obtained and grafted by electrochemical reduction of the 1,8-bis naphthalenediazonium salt, as presented in Figure 6 [40].

*Other 2D materials.* Due to the interest in graphene, other 2D materials were modified with diazonium salts.

Few layers of black phosphorous were modified with a zinc phthalocyanine-based diazonium salt for applications in non-linear optics [41].

**Figure 7:**
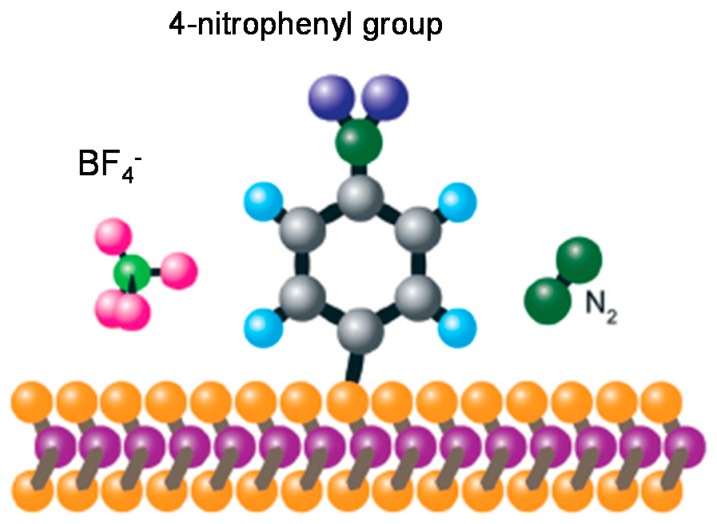
Modification of a MoS_2_ sheet by 4-nitrobenzenediazonium (Mo violet, S orange). From Reference [42] with permission of the Royal Society of Chemistry.

Metal dichalcogenides MX_2_, where M is a transition metal (M = Mo, W, Nb, Ta, etc.) and X is a chalcogen (X = S, Se, or Te), were exfoliated into two-dimensional (2D) nanosheets. Among them, MoS_2_, WS_2_, MoSe_2_, and WSe_2_ were modified with diazonium salts (Figure 7). The pnictogen chalcogenides Sb_2_S_3_ and Bi_2_S_3_ were exfoliated into one-dimensional (1D) nanoribbons and 2D nanosheets and derivatized [42].

MXenes are 2D nitrides and carbides; for example, Ti_3_C_2_ was intercalated by Na^+^ ions and then grafted by reaction with 4-sulfonylbenzenediazonium to obtain enhanced super-capacitive performances [43].

*Nanoobjects.* Nanoparticles were capped with aryl groups through diazonium chemistry to imbue these objects with new properties such as catalysts, scavengers, and reagents [44]. It is also possible, with the same reaction, to decorate various surfaces with nanoparticles.

*Nanoparticles can be stabilized.* Iron oxide Fe_2_O_3_ nanoparticles could be capped with BF_4_, N_2_-C_6_H_4_-(CH_2_)_2_-OH by spontaneous reaction in basic aqueous medium. These nanoparticles retained their magnetic properties and were soluble in apolar organic solvents such as dichloromethane, tetrahydrofurane (THF), toluene, and chloroform, as well as in polar solvents such as methanol, ethanol, or water [45,46]. Cerium oxide (CeO_2_) nanoparticles were grafted with 4-methyl-, 4-ethyl-, and 4-*n*-butyl benzenediazonium; the water contact angle increased from 34° to 63° and 125° as the chain length increased. With this modification, the cerium oxide NPs are more compatible with an electrolytic solution for the formation of coatings or a metal composite matrix [47].

*Nanoparticles can be used as catalysts*. TiO_2_ nanoparticles were modified with 4-diphenylamine groups (DPA) by reaction of the corresponding diazonium salt; from this surface, polyaniline (PANI) was prepared by in situ polymerization of aniline. This bonding prevented polyaniline from leaching in polar solvents. This TiO_2_–DPA–PANI assembly efficiently catalyzed the degradation of the dye methyl orange in aqueous media under UV light [48].

**Figure 8:**
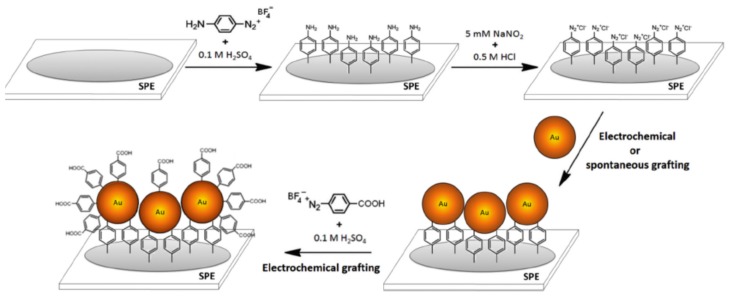
Gold nanoparticles covalently attached to a screen-printed electrode (SPE) and modified with caboxyphenyl groups for sensing metal ions. From Reference [49] with permission from Elsevier.

*Nanoparticles can also be attached to surfaces* for increasing analytical sensitivity. Gold nanoparticles were attached to the surface of screen-printed electrodes (SPE) derivatized with aminophenyl groups; the amino group was transformed into a diazonium that reacts with gold nanoparticles. In turn, these nanoparticles were modified by reaction of 4-carboxybenzenediazonium. With this system, it was possible to detect Pb(II) down to 2.5 × 10^−9^ M (Figure 8) [49].

### 1.5. The Surface Aryl Bond

One of the most important features of diazonium grafting is the stability of the construct; this was demonstrated by several methods including (i) thermogravimetry, where exfoliated graphene lost only 7% of its coating at 400 °C [50], (ii) spectrometric methods, where a small Raman band at 412 cm^−1^ was assigned to the *Au(nP)*–C(aryl) bond of gold nanoparticles modified by 4-nitrobenzenediazonium [51]; grafting of an aryl group on graphene [52] and carbon nanotubes [53,54] transformed an *sp*^2^ carbon into a *sp*^3^, which translated into the growth of the D-band.

### 1.6. The Structure of the Grafted Film

**Figure 9:**
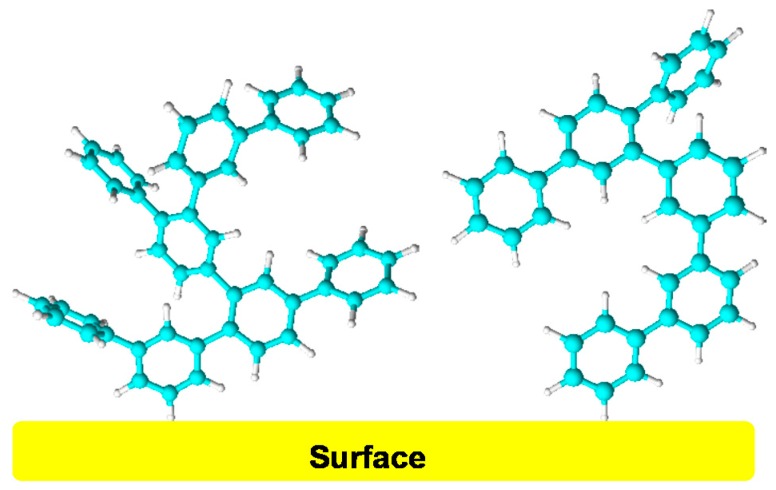
A schematic presentation of a film obtained from diazonium salts.

The structure of the films obtained by dediazoniation of diazonium salts is quite complex and is not yet been completely elucidated. Upon dediazoniation, radicals are responsible for the grafting reaction and for the structure of the obtained nanometric films. The radicals react on the surface but also on the first grafted groups to produce “multilayered” films. This term is widely but somewhat abusively used in the literature as the structure of the film is not layered as, for example, layer-by-layer constructs. Figure 9 presents a very schematic presentation of a film obtained from diazonium salts.

Aryl–aryl bonds permit a free rotation; optical absorption spectroscopy of thin (1–15 nm) oligomeric polyaromatic films attached to an atomically flat pyrolyzed photoresist film (PPF) permitted concluding that the molecular layers were composed of *n*-mers possessing very limited conjugation that extended only to one monomer [55]. However, by combining the electrografting of diazonium salts on Au and the oxidative electropolymerization of biphenyl in an ionic liquid, a regular poly(*para*-phenylene) film was obtained [56].

**Figure 10:**
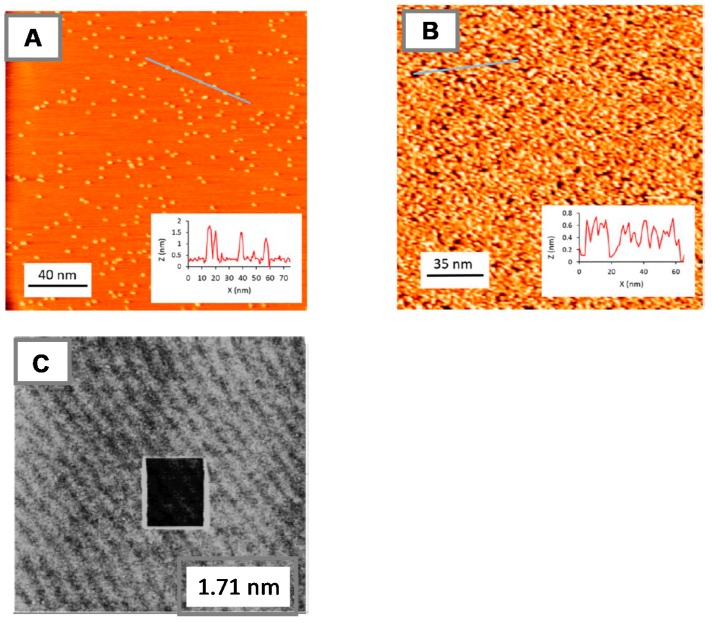
(**A**,**B**) STM (scanning tunneling microscopy) images of films obtained on highly oriented pyrolytic graphite (HOPG) after spontaneous grafting of (**A**) 4-nitrobenzenediazonium and (**B**) 3,5-bis-*tert*-butylbenzenediazonium salts. (**C**) AFM (atomic force microscopy) image of a monolayer obtained from 4-nitrobiphenyldiazonium salt (l = 1.21 nm) on PPF, a near-atomically flat carbon; in the black square, the layer was erased by AFM scratching. From References [57,58] with permission of the American Chemical Society.

The thickness of diazonium derived films is measured by ellipsometry, AFM (atomic force microscopy), or STM (scanning tunneling microscopy). STM images (Figure 10A) showed a growing film of 4-nitrophenyl groups on the surface of HOPG; one can observe isolated oligomeric groups (up to 2 nm), indicating that, on this surface, the aryl radical reacts faster on the first grafted group than on the surface [57]. On the contrary, on PPF, a uniform monolayer was obtained [58], indicating that the reaction is faster on PPF than on the first grafted group (Figure 10C). This is related to the difference in reactivity of the two different carbons. This renders the control, a priori*,* of the thickness quite difficult.

Thick films up to ~100 nm were obtained by electrografting 4-nitrobenzenediazonium at the reduction potential of the nitrophenyl group [59]. At this potential, the radical anion of the nitrophenyl group was formed, and electrons could transfer through the film, reach the surface, and reduce a diazonium cation; this process permitted the thickening of the film.

Conversely, many efforts were devoted to the formation of monolayers. Indeed, such monolayers would be very useful for the preparation of biosensors as they should provide faster and uniform electron transfer to a bioreceptor. Figure 10B shows the STM image of a monolayer (thickness ~0.6–0.8 nm) of 3,5-bis-*tert*-butylbenzenediazonium [57]. The steric hindrance of the two bulky *tert*-butyl groups prevented the aryl radicals from reacting of the aromatic ring and, consequently, the growth of the film [60]. However, with this method, post-modification was not possible; later on, it was modified to permit further reactions on the film [61].

A more general method [62] involves electrografting in the presence of redox mediators. Monolayers (0.6–0.9 nm) of 4-bromo, 4-iodo, 4-methoxy, and 4-diethylamino phenyl groups were obtained in the presence of three redox mediators: 2,2-diphenyl-1-picrylhydrazyl, chloranil, and dichlone. The efficiency of the method rests on a fast redox cross-reaction in the diffusion layer between the diazonium compound and the reduced form of the selected inhibitor. This method should permit preparing, in a repetitive manner, reactive monolayers that would be useful for biosensors due to their fast electron transfer.

**Figure 11:**
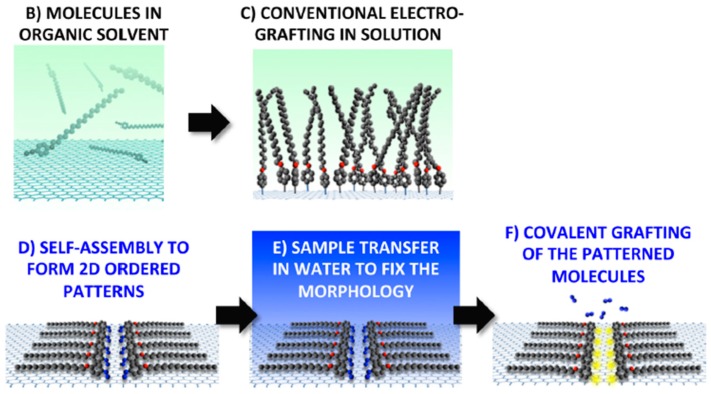
Adsorption and electrografting of long-chain benzenediazonium tetrafluoroborate on graphene. From Reference [63] with permission of the American Chemical Society.

Disordered oligomeric films without regular patterns on the surface were obtained from diazonium salts [2]. Using high-quality graphene and a diazonium with a long alkyl chain (C_22_H_45_-O-C_6_H_4_N_2_^+^ BF_4_^−^) [63], it was possible to obtain patterns of adsorbed molecules (imaged by STM); however, when the diazonium salt was reduced, grafting occurred and a new pattern was obtained. In this way, the authors obtained a pattern of grafted molecules; it is, however, surprising that the thickness of the grafted pattern was only 2 nm. As the diazonium was *para*-substituted, the molecules should have been more or less vertically aligned on the surface *sp*^3^ carbons (Figure 11).

## 2. Applications to Biosensors

A biosensor is composed of a bioreceptor (probe), which selectively binds the analyte of interest (target), and a transducer, allowing the transformation of the probe/target recognition event into a physical signal. Research and development of biosensors is extensively studied because they permit easy, rapid, low-cost, highly sensitive, and selective detection of analytes. They allow ultrasensitive point-of-care detection of markers for diseases and should lead to advances for next-generation medicinal applications such as personalized medicine. In addition to biomedical applications, biosensors are also capable of responding to the current needs for environmental monitoring. For both fields of applications, the ongoing trend is miniaturization, parallelization, and integration of sensors into everyday objects. However, despite the intense research and development activities around biosensors, very few of them actually reached the market because of their non-optimal performances [64].

The sensor selectivity, sensitivity, and robustness (stability, reproducibility, etc.) are mainly controlled by the intrinsic bioreceptor characteristics, such as its affinity toward the target, as well as its stability in the sensor operating conditions. In a biosensor, the bioreceptor is usually grafted onto a surface, i.e., in the close vicinity of the transducer. Hence, sensor sensitivity and robustness depend also on the methodology deployed for the bioreceptor immobilization onto the transducing surface. The accessibility of the target to the recognition site of the biomolecule, the grafting stability, and the distance between the receptor and the transducer (surface) are all parameters to be optimized in order to improve the device analytical performance. All these parameters depend on the method chosen to immobilize the biomolecule. Historically, conducting polymers (CP) were widely used as a conductive matrix to produce affinity (DNA, proteins, etc.) or enzymatic electrochemical sensors. The possibility of inserting the biomolecule inside the transducer material (CP) led to many ultrasensitive enzymatic sensors. However, CPs do not allow thin-film production, leading to sensors showing long response times (long delays to reach a stable signal). The other widely used approach is to immobilize the bioreceptor by self-assembly, including chemisorption of thiols. This approach led to the development of complex (controlled immobilization of several bioreceptors) and ultrathin (few nm between the binding site and the transducer) sensing systems presenting excellent analytical performance in terms of sensitivity. However, critical disadvantages remain, such as (i) the weak stability of the metal–S bond, (ii) the limited number of substrates (essentially noble metals), and (iii) the difficulty of localizing the film deposition. Indeed, in the development of chips comprising several sensors, it is necessary to have a functionalization method for addressing the deposition step. In this respect, electrodeposition is definitely advantageous as it offers the possibility of functionalizing specific (polarized) zones directly by the bioreceptor or an anchoring function thereof.

Electroreduction of diazonium salts is a rarely used functionalization method compared to CP and self-assembly, despite the achievement of extremely stable surface modifications (covalent bonding) that contribute to the stability of the biosensor. In addition, the high reactivity of the diazonium function allows a fast and extremely dense grafting on a wide range of substrates.

Indeed, the interest in the diazonium salt electroreduction approach is largely related to the remarkable reactivity of the diazonium function. However, its limited use in the field of biosensors is probably due to the same reason. Indeed, this method generally leads to “multilayered” structures that may increase the bioreceptor/surface (transducer) distance and, therefore, potentially provoke a loss of sensitivity (issue 1). Moreover, in the case when the biomolecule itself is modified by a diazonium salt, to ensure high affinity, it must be oriented with respect to the surface (accessibility to the target, structural reorganization associated with recognition, etc.) and not denatured upon grafting. The high reactivity of diazonium groups can lead to a random distribution and orientation of the receptors (issue 2), as well as to their degradation (issue 3); the diazonium group attached to, for example, a protein can react with a wide range of biological functional groups (phenols, amines, etc.) of the same protein or another molecule. These issues need to be completely addressed in order to take advantage of diazonium chemistry for the realization of biosensors.

In order to bind the receptor, an aryldiazonium group must be firstly equipped; this is generally achieved by peptidic coupling. Conversely, a platform can be created by reacting the surface with a diazonium, including a 4-substituent designed to react with the bioreceptor.

The many reviews (Table 1) published for the construction of biosensors using diazonium salts as anchoring molecules testified to the interest in this method [7,8,65,66,67,68,69]. The objective of this section is to carry out a review of biosensors using the electroreduction of diazonium salts structured by analyte type, each of them having specificities in terms of sensor typology and expected analytical performances. For each target, any progress made to address the aforementioned issues is highlighted.

### 2.1. Detection of Small Molecules of Biological Interest

*Glucose.* The detection of glucose is a challenge related to diabetes; a number of papers were published dealing with the detection and quantification of this molecule through diazonium chemistry. Note that the normal glucose content of blood is from 3.9 to 7.1 mmol/L (70 to 130 mg/dL). However, on the one hand, blood samples require the use of several membranes to avoid sensor biofouling, and, on the other hand, current glucose monitoring approaches target tears or sweat as glucose vectors in order to avoid body penetration. Therefore, accurate continuous glucose monitoring devices need a concentration linearity range in the micromolar range.

**Figure 12:**
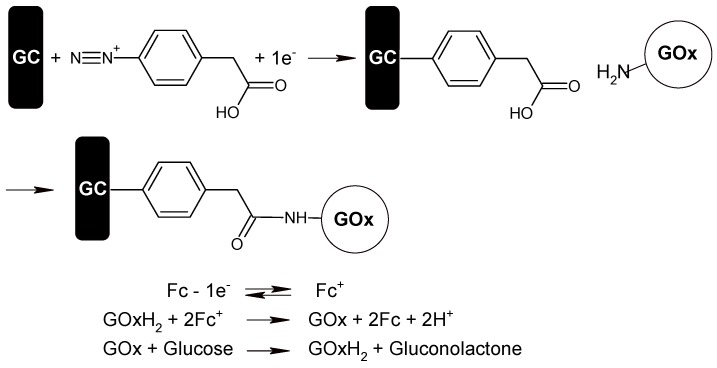
Detection of glucose by attaching glucose oxidase (GOx) to a glassy carbon surface. Catalytic scheme for the electro-catalyzed oxidation of its reduced form (GOxH_2_).

Glucose is electrochemically detected through its oxidation into gluconolactone; this reaction is catalyzed by glucose oxidase (GOx), which is reduced to its reduced form (GOxH_2_), which is then reoxidized either directly by electron transfer from the electrode or more often through a mediator such as ferrocene (Fc in Figure 12). In agreement with the subject of this review, we describe the experiments where GOx is attached to the surface of the electrode by diazonium chemistry. Table 2 gives an overview of the different papers published on the subject. This table is divided into two parts: electron transfer through a mediator or direct electron transfer.

As an example of the methods involving a mediator, we present the first paper describing the use of a diazonium salt for detecting glucose. It involved the attachment of glucose oxidase to a glassy carbon surface modified with 4-phenylacetic acid diazonium fluoroborate through carbodiimide coupling [73]. Glucose was detected (Figure 12) through the electrochemical signal of the ferrocene/ferricinium–methanol couple (Fc/Fc^+^) acting as an electron shuttle between the electrode and GOx/GOxH_2_. Based on the catalytic regeneration of ferrocene, it was possible to determine the surface concentration of the active enzyme as Γ ~1.8 × 10^−13^ mol cm^−2^, about one-tenth of the estimated value of a monolayer of GOx (Γ ~1.7 × 10^−12^ mol∙cm^−2^). A similar system was constructed from *trans*-cinnamic acid, showing good selectivity for the various possible compounds interfering in glucose analysis, namely, ascorbic acid and 4-acetamidophenol [74].

**Figure 13:**
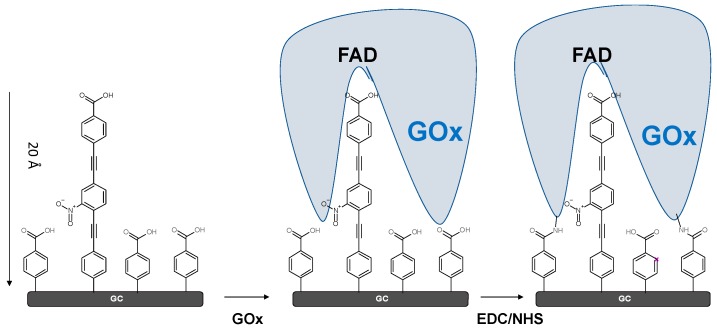
Biosensor of glucose based on the direct electron transfer to the of GOx flavine adenine dinucleotide (FAD) co-enzyme through a molecular wire.

Direct electron transfer from the electrode to a GOx enzyme is also possible by diazonium chemistry but requires fine control of the overall structure. Indeed, to obtain an efficient enzyme wiring, a bottom-up approach needs to be implemented. The active center of GOx is a flavine adenine dinucleotide (FAD) buried deep inside the pocket of a proteinic structure of the enzyme. Gooding et al. designed a molecular wire (a 20-Å-long oligo(phenylethynyl)) able to reach the FAD active site and providing a fast electron transfer to GOx [75]. In addition, these bonded molecular wires were diluted in 4-carboxyphenyl groups (30/1) that served the twin purposes of being a spacer between molecular wires and an anchor to maintain the attached GOx on the surface via covalent peptidic coupling (Figure 13). The surface coverage of active GOx was calculated to be 2.41 × 10^−12^ mol∙cm^2^, and the rate of electron transfer was k_ET_ = 78 s^−1^.

**Figure 14:**
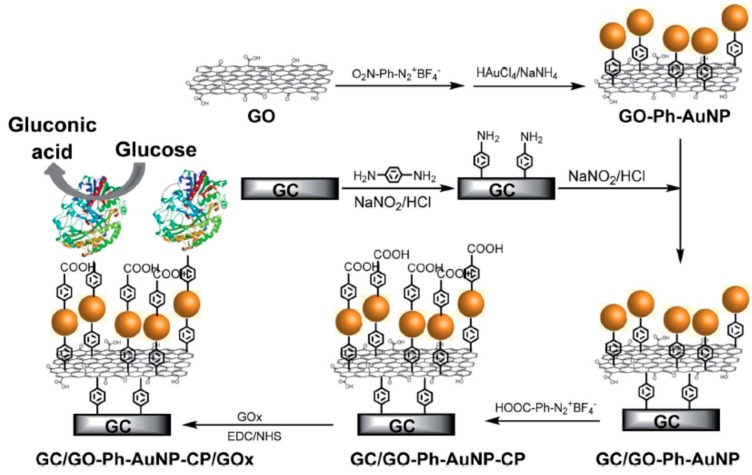
Attachment of glucose oxidase through graphene oxide and gold nanoparticles. From Reference [76] with permission of the Royal Chemical Society.

Another complex assembly is presented in Figure 14, where grafting of diazonium salts was used for attaching (i) gold nanoparticles to graphene oxide (GO), (ii) modified GO to the glassy carbon (GC) electrode, and finally (iii) GOx to gold nanoparticles [76]. These two examples underline the ability of diazonium chemistry to form complex (nano)structures similar to those obtained by self-assembly.

Table 2 gathers the different architectures for glucose biosensors obtained through diazonium salt chemistry.

*NAD^+^/NADH*. The nicotinamide adenine dinucleotide redox co-factor, NAD^+^, is the coenzyme of over 300 dehydrogenase enzymes (e.g., lactate dehydrogenase, alcohol dehydrogenase, glucose dehydrogenase). The quantitative detection of the reduced form, NADH, can be used as a measure of enzymatic activity. Amperometric biosensors based on this strategy were developed to assay of the corresponding enzymatic substrate molecules (e.g., lactate, malate, and ethanol).

NADH was detected [87,88,89,90] on an array of five electro-addressable electrodes. All the electrodes were grafted with 4-nitrophenyl groups; then, on two of these electrodes, the nitrophenyl group was reduced to aminophenyl to which pyrroloquinoline quinone was bonded by peptidic coupling. This quinone acts as a mediator for the oxidation of NADH [87]. In a similar way, anthraquinone, [89] toluidine blue [88], and azure A (for the detection of ethanol) [90] were attached to electrodes.

*Other biomolecules.* Some sensors were described that, based on diazonium salts, can detect different drugs; for example, ranitidine, a histamine H2 receptor antagonist, was detected on aminophenyl-modified gold nanoparticle films deposited on a GC electrode by differential pulse voltammetry (DPV) [91]. Calcitonin, a tumor marker, was detected on a GC electrode modified by 4-carboxy or 4-nitrobenzenediazonium and attachment of gold nanoparticles and graphene oxide; the increased surface area of the immunosensor translated into an enhanced sensitivity [92]. Uric and ascorbic acids were also detected by diazonium-based sensors [93,94,95]. For example, uric acid was detected with an Au gate field-effect transistor (FET)-based sensor where the gold surface was modified by a monolayer of 4-nitrobenzenediazonium in the presence of DPPH (2,2-diphenyl-1-picrylhydrazyl) [95]. Estradiol was detected by attachment of an aptamer (NH_2_-APT) to a GC-reduced graphene oxide surface modified by reaction of 4-carboxybenzenediazonium [96].

*Toxins.* Biosensors were developed against dangerous toxins. Aflatoxins are- highly toxic mycotoxins produced by fungi. Among them, AFM1 can be found in commercially available milk; it is, therefore, considered as one of the most serious problems of food safety, and the level of aflatoxin in milk is subject to safety regulations. Okadaic acid is one of the most common marine biotoxins, which is ingested through filter feeding mechanisms by various species of shellfish such as mussels. Ochratoxin A is a carcinogenic mycotoxin that was identified as a contaminant in cereals, coffee, cocoa, dried fruits, and pork. Therefore, electrochemical detection of these toxins is important, and biosensors were constructed either via modifying a surface by attaching antibodies and aptamer or via modifying the toxin itself and attaching the modified species (Table 3).

**Figure 15:**
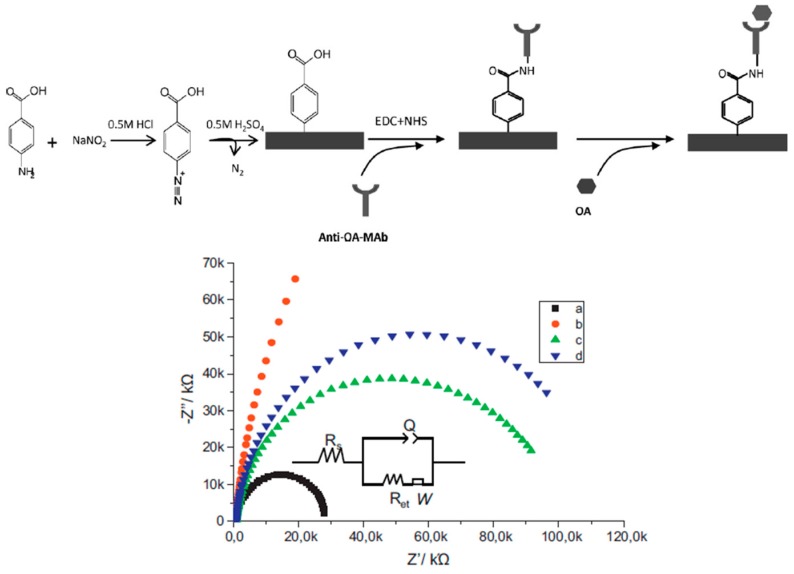
A schematic presentation of the surface chemistry used for immobilization anti-okadaic acid (OA) monoclonal antibody on an SPE electrode, and Nyquist plots of 1 mM [Fe(CN)_6_]^4−/3−^ for (**a**) bare SPE, (**b**) SPE modified by 4-carboxybenzenediazonium, (**c**) and an antibody, (**d**) in the presence of 10 µg/L ochratoxin. The insert is the equivalent electric circuit applied to fit the impedance spectroscopy. From Reference [98] with permission of Elsevier.

For example, okadaic acid was recognized by its attached antibody and detected by Electron Impedance Spectroscopy (EIS), as presented in Figure 15.

*Biogenic amines.* Biogenic amines are synthesized and degraded during normal metabolism of animals, plants, and microorganisms. Histamine, putrescine, cadaverine, tyramine, tryptamine, spermine, and spermidine are considered to be some of the most important biogenic amines in food. They were detected by attaching monoamine oxidase to the surface of SPE detection in the presence of ferrocene methanol as a detector [102].

### 2.2. Detection of Polypeptides and Proteins

Many polypeptides, proteins, and enzymes are important in biological or medical processes. Their detection and quantification in very minute quantities in body fluids is necessary; this can be achieved by using diazonium salts that provide an anchor to attach proteins to surfaces where they are detected. Two methods were used: (i) the protein is modified with an aminophenyl group (mainly by peptidic coupling), and this aminophenyl modified protein is then transformed to a diazonium salt that is attached to the surface; (ii) a diazonium salt with an appropriate 4- substituent (mainly carboxylic and amino groups) is attached to the surface and further reacts with the protein. These two methods (Table 4) permit creating a diazonium-based sandwich immunoassay; an analyte (most often a protein) is detected through the use of a diazonium-anchored antibody (also termed immunoglobulin IgG) (or antigen), and the assay is completed by attaching a detectable group to this construct (for example, by luminescence). Table 4 gathers examples of such biosensors.

**Figure 16:**
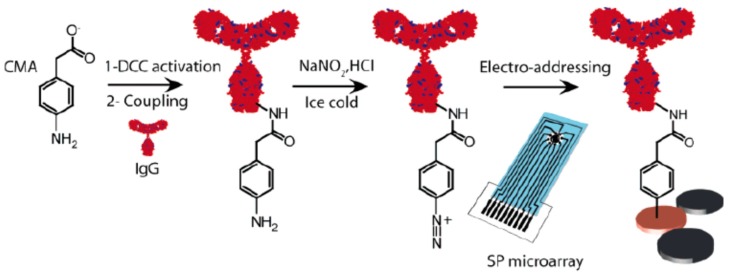
Electrografting of an immunoglobulin G (IgG) on an array of screen-printed electrodes. From Reference [104] with permission of Wiley.

The first examples of such diazonium-based immunoassays were published by Marquette [103,104]; an array of individually addressable screen-printed electrodes was modified with an IgG. This was achieved by (i) coupling the carboxylic group of 4-carboxyaniline to an amino group of IgG by peptidic coupling (DCC/NHS, N-hydroxysuccinimide N,N′-dicyclohexylcarbodiimide), (ii) diazotizing the pending amino group in acidic water (20 mM HCl and 20 mM NaNO_2_), and (iii) electrografting the diazonium-labeled IgG to a connected electrode of the array. This electrografting was characterized, as for other diazonium salts, by a decrease of the drawn-out wave of the diazonium salt upon repetitive scanning. This reaction sequence is presented in Figure 16.

**Figure 17:**
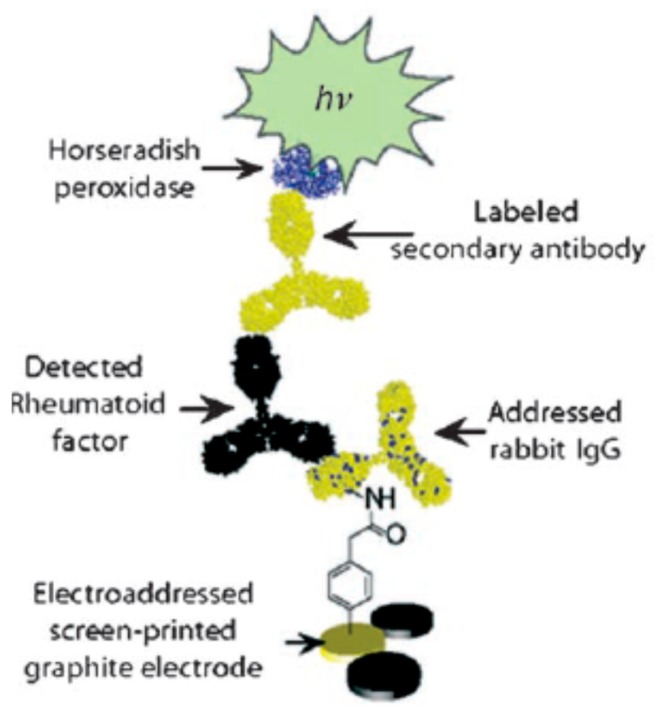
A sandwich immunoassay for the detection of rheumatoid factor. From Reference [104] with permission of Wiley.

The final step of the assembly is presented in Figure 17 in the case of a rheumatoid factor (a family of human antibodies largely involved in rheumatoid diseases) that binds to the surface-attached IgG. The detection was achieved by binding a secondary antibody labeled by a horseradish peroxidase; this final enzyme catalyzed the oxidation of luminol with light emission at 428 nm. Therefore, detection of this emission permitted quantifying the presence of rheumatoid factor in human serum in the range 5.3–485 IU∙mL^−1^. Detection was also achieved on SPRi (surface plasmon resonance imaging) surfaces by direct reflectivity change.

**Figure 18:**
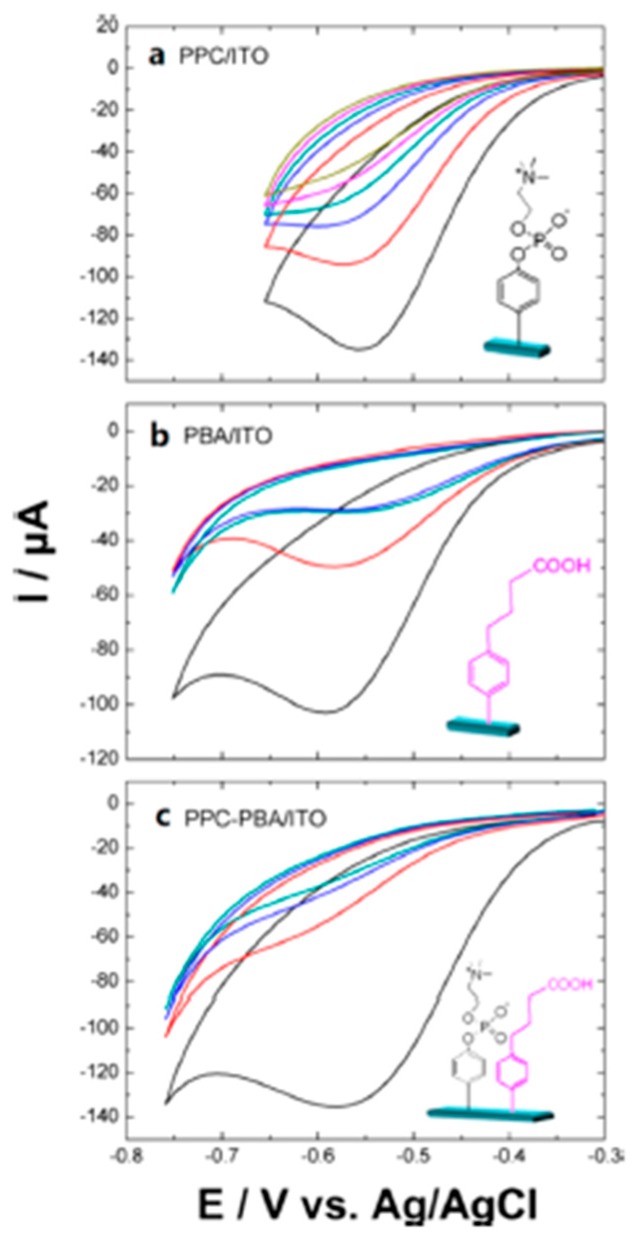
Cyclic voltammetry of the diazonium salts of 4-aminophenyl phosphorylcholine (PPC), 4-(4-aminophenyl) butyric acid (PBA), and their mixture on an indium tin oxide (ITO) electrode. From Reference [117] with permission of the American Chemical Society.

The second strategy involves the fabrication by diazonium chemistry of platforms that can be connected to proteins. An excellent example was provided by a paper of Gooding that described the detection of tumor necrosis factor α (TNF-α) in whole blood [117]. TNF-α is a typical early-stage indicator of an inflammatory reaction, in response to infection or cancer. Affinity biosensors are difficult to operate in whole blood because biofouling of electrode surfaces compromises the performance of the final device. To prevent this phenomenon, a platform was prepared on ITO by reduction of two diazonium salts derived from 4-aminophenyl phosphorylcholine (PPC) and 4-(4-aminophenyl) butyric acid (PBA). Therefore, this mixed surface comprised phosphorylcholine groups that prevented biofouling of the electrode and phenylbutyic acid groups that permitted the attachment of antibodies as biorecognition elements. Other anti-biofouling molecules could be attached to electrode surfaces such as polyethyleneglycol molecules, but these types of long-chain molecules give rise to passivated surfaces with high impedance. Upon electrochemical reduction, the diazonium salts of PPC (E_p_ = −0.55 V/(Ag/AgCl) and PBA (E_p_ = −0.58 V/(Ag/AgCl), as well as their mixture, gave rise to the typical pattern of diazonium salts, where the current decreases upon repetitive scanning, as shown in Figure 18.

**Figure 19:**
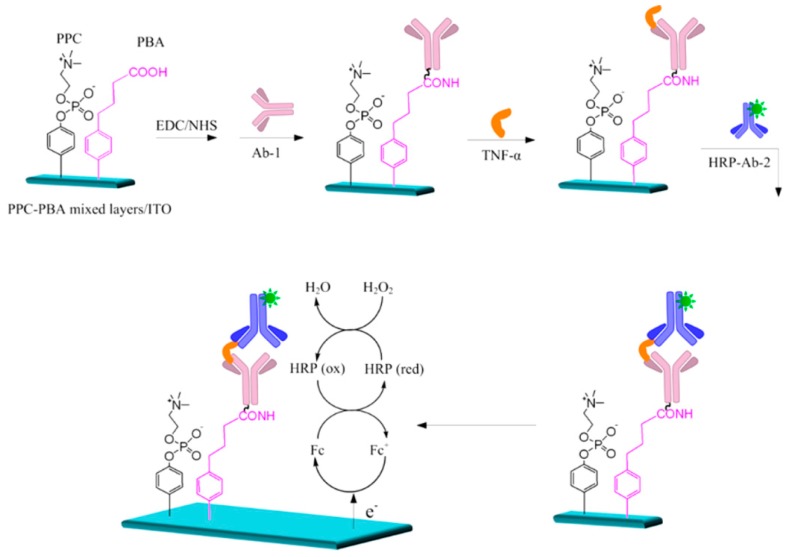
Immunoassay of tumor necrosis factor α (TNF-α) factor based on a mixed antifouling platform. From Reference [117] with permission of the American Chemical Society.

These modified ITO electrodes were characterized by XPS (X-ray Photoelectron Spectroscopy), cyclic voltammetry of redox probes (Fe(CN)_6_^3−/4−^), and EIS. The immunosensor was on this platform as presented in Figure 19. The capture antibody (Ab1) was immobilized onto the PPC–PBA/ITO surface via the classical EDC/NHS (EDC: 1-éthyl-3-(3-diméthylaminopropyl)carbodiimide) conjugation reactions between COOH groups on the mixed layer surface and residual amino groups of the Ab1. The final steps involved the binding of the analyte TNF-α and finally of the HRP-conjugated detection antibody. The consumption of H_2_O_2_ by HRP was detected by amperometry, and TNF-α concentrations in the range of 0.01–500 ng/mL were detected. The interference of human serum albumin or hemoglobin was limited by the presence of phosphorylcholine on the surface [117]. This example illustrates the possibility of realizing mixed layers with controlled structures. This new approach enhances the analytical performance by implementing both a bioreceptor and a non-specific adsorption reducer. Prior to this publication, only thiol-type self-assembly and co-polymerization approaches were used to obtain such structures.

**Figure 20:**
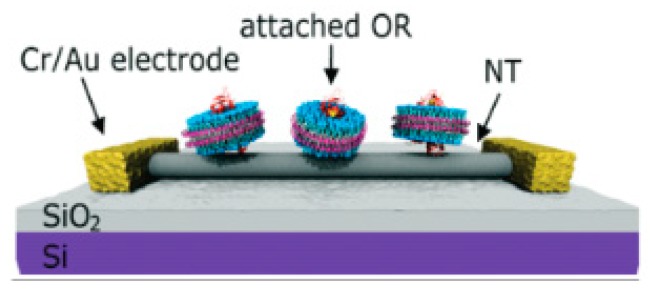
Transistor for the detection of odors (“e-nose”). From Reference [112] with permission of the American Chemical Society.

An “e-nose” capable of detecting different odors (eugenol, *n*-amylacetate, etc.) was constructed as shown in Figure 20 by coupling mouse olfactory receptor proteins (ORs) with carbon nanotube transistors. The CNT was modified with 4-carboxybenzenediazonium, and the ORs were attached by peptidic coupling. The resulting devices transduced signals associated with odorant binding to ORs in the gas phase under ambient conditions [112].

### 2.3. Detection of DNA

Affinity sensors using nucleic acids as bioreceptors are probably the most common biosensors. Indeed, nucleic acids are used to bind another nucleotide sequence (DNA, RNA, microRNA (miRNA), etc.) or, in the case of aptamers, a different type of target (e.g., small organic molecules, metal cations, proteins, etc.). Biosensors including a diazonium electroreduction step in their fabrication process already proved their relevance for DNA sensing. Methods for recognition of DNA sequences were as follows: (i) the DNA sequence to be recognized was equipped with a diazonium salt, electrografted and recognized in different ways [103]; (ii) the target DNA sequence was bonded to the surface through avidin–biotin recognition [122] or another method [123]; (iii) the target DNA sequence was linked to the surface and recognized by its complementary sequence [124,125]; (iv) a symmetric reaction where the complementary sequence was bonded to the surface was also possible [126]. The detection was achieved either by fluorescence or by electrochemistry (DPV, EIS) (Table 5).

A mixed layer obtained from two diazonium salts (4-carboxybenzenediazonium + the diazonium salt of an amino derivative of 5-hydroxy-1,4-naphthoquinone, juglone) was prepared. The carboxylic group was activated by EDC/NHS, and a primary amine-functionalized DNA strand (NH_2_-DNA probe) was coupled to the carboxylic acid. This attached DNA sequence recognized its complementary chain equipped with a fluorescent label, but hybridization was also detected by 3AC) voltammetry of the naphthoquinone group [128].

**Figure 21:**
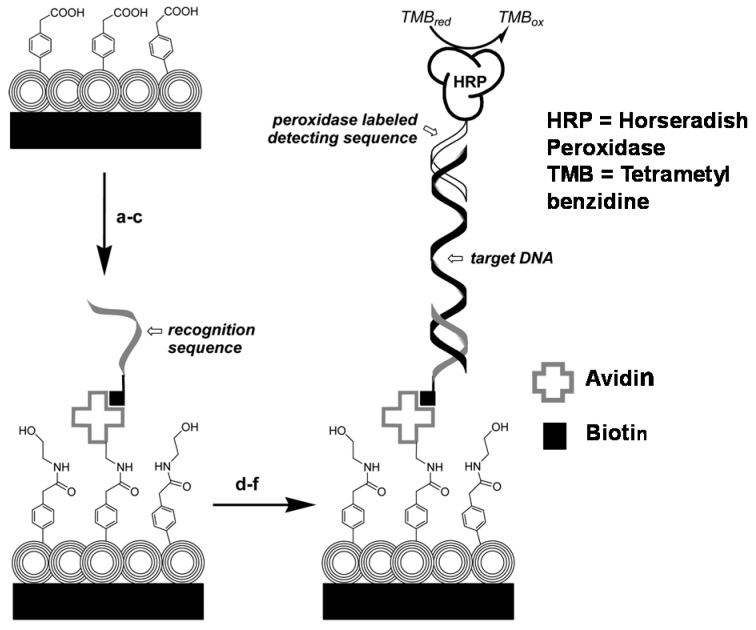
DNA sensors for human papillomavirus oncogene detection. From Reference [130] with permission of the American Chemical Society.

Human papillomavirus is a DNA virus responsible for cervical cancer. It can be detected based on the sequence of reactions presented in Figure 21. Carbon nano-onions are multilayered fullerenes concentrically arranged one inside the other; they were deposited onto a GC surface to form a stable micrometric film. This carbonaceous film was modified by the diazonium salt of phenylacetic acid. The COOH groups of the surface were activated using carbodiimide chemistry, followed by covalent immobilization of streptavidin and incubation of a biotinylated DNA capture probe (Figure 21a–c). Hybridization was accomplished with the target DNA sequence. Finally, an HRP-labeled reporter probe was introduced, and amperometric detection of the oxidation of tetramethylbenzidine permitted quantifying the analyte down to 0.11 μA∙nM^−1^. Incorporation of carbon nano-onions on the surface resulted in better sensitivities and lower limits of detection than unmodified GC due to the increased surface area [130].

### 2.4. Cells

**Figure 22:**
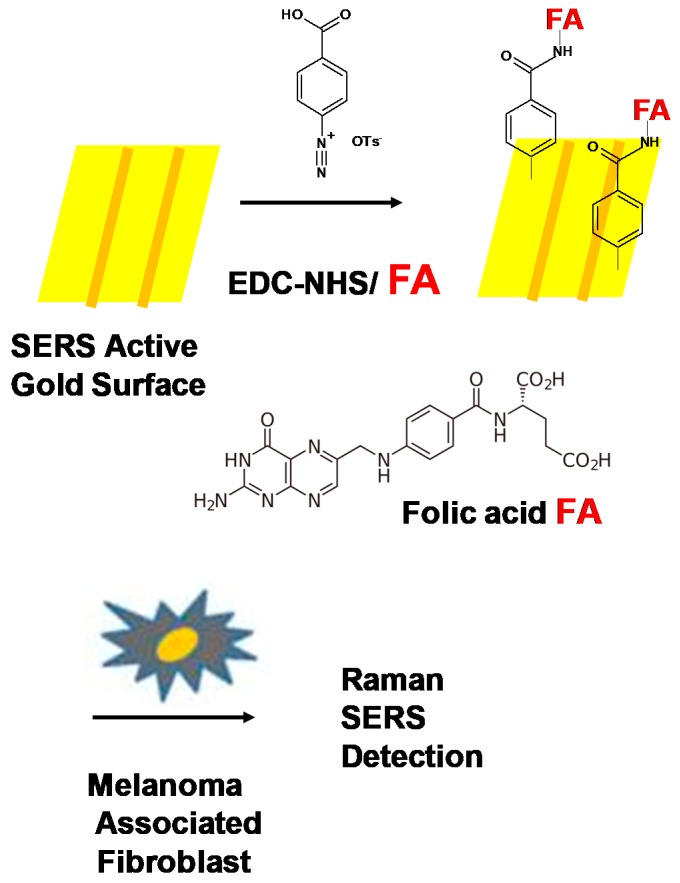
Detection of normal and melanoma-associated cell secretome by SERS (surface-enhanced Raman spectroscopy).

Gold SERS (surface-enhanced Raman spectroscopy) active substrates were modified with 4-carboxyphenyl groups, and the carboxylic groups were coupled to amino groups of folic acid. With such a surface, it was possible to test the presence of normal and melanoma-associated cells in a cultivation medium. The SERS spectra of the folic acid modified surface indicated the presence of differences arising from the interaction of the bio-liquid with the functional surface [133] (Figure 22).

## 3. Concluding Remarks

The large and growing demand for robust and reliable detection devices continues to motivate much work around biosensors. Given the advantages of the electroreduction of diazonium salts in terms of stability of (bio)molecular buildings, it is very likely that this method will contribute to improving the reliability and stability of detection systems. However, before being able to take full advantage of diazonium chemistry, attention must be paid to reach a finer control of film thickness and composition, which is the current main challenge. Recent advances in controlling the thickness and composition of mixed layers obtained by reduction of diazonium salts are the first steps toward the realization of covalent buildings of controlled architecture. Beyond the classical approaches of biodetection, there is a tendency to integrate biosensors into everyday objects through printing methods. The reactivity of the diazonium function makes it possible to consider, as a future prospect, the development of a new generation of functional inks able to bind covalently to the surface of the substrate or to an underlying layer. This capability would allow the integration of a variety of functions, including biosensing ones, under ambient conditions over a wide range of surface areas.

## Figures and Tables

**Table 1:** Reviews on biosensors built with the help of diazonium chemistry.

[1]	Some of advantages and disadvantages of alkanethiol and diazonium salts for the modification of electrode surfaces, and selected examples
[66]	The use of diazonium salts as surface modifiers and coupling agents, as well as some applications in biosensing
[67]	A general overview of biosensors including different methods that permit attaching sensing groups to a surface
[68]	A review of the author’s work concerning biosensing of antibodies, oligonucleotides, and enzymes onto conductive supports
[69]	Advances in the use of aryldiazonium salts for modifying interfaces in sensors and biosensors
[70]	A review that outlines the potential of diazonium chemistry to prepare single or multianalyte electrochemical affinity biosensors on screen-printed electrodes (SPEs)
[71]	A review that evaluates the methods through which redox proteins can be attached to electrode surfaces in a “wired” configuration that facilitates direct electron transfer
[72]	Copper(I)-catalyzed click chemistry as a tool for the preparation of electrochemical (bio)sensors

**Table 2:** Glucose sensors based on diazonium grafting. GOx—glucose oxidase.

Surface ^(a)^	Attached Aryl Group		Characteristics c = Concentration Range Γ (GOx) mol∙cm^−2^, k_ET_ T = Turnover S= Sensitivity	Reference
		*Mediator*		
GC	4-phenylacetic	Fc-CH_2_OH ^(b)^	Γ ~1.8 × 10^−13^	[73]
GC	4-phenylcinnamic	Fc-CH_2_OH ^(b)^	-	[74]
GC	4-phenylacetic + layer by-layer ^(c)^	Fc-CH_2_OH ^b)^	Γ ~1.1 × 10^−12^	[77]
Aligned CNT	4-aminophenyl ^(d)^	Pt nanoparticles	c = 1 × 10^−2^–7 mM Stability 20 days	[78]
CNT	4-(2-aminoethyl) phenyl	Fc-CH_2_-CH_2_-COOH ^(e)^	c = 5–50 mM s = 0.83 µA mM^−1^	[79]
GC	Nile blue	O_2_^f)^	c = up to 2.5 mM	[80]
GC ^(g)^	4-nitrophenyl	Fe(CN)_6_^3−/4−^	c= up to10 mM	[81]
Pt ^(h)^	4-fluoro 4-methyl 4-methoxy	Fc-CH_2_OH	c = 0.2–10 mM In the presence of ascorbic acid and uric acid	[82]
Porous HOPG ^(i)^	To create the porosity	Fc-CH_2_OH	5 µM–100 mM	[83]
		*Direct Electron Transfer*		
GC-Ar-GO-nP	4-carboxyphenyl	Direct electron transfer to GOx	C = 0.3–20 mM k_ET_ = 8.3 s^−1^ T = 112 s^−1^.	[76]
GC	4-carboxyphenyl + oligo(phenylethynyl) ^(j)^	Direct electron transfer to GOx	0–25 mM T = 1.1 s^−1^	[75]
GC	3-phenylboronic	Direct complexation of GOx	−50 mM	[84]
GC + GO ^(k)^	Thionine	Electrostatic adsorption of negatively charged GOx on positively charged functions of thionine	c = 0.5–6.0 mM s = 43.2 mA mM^−1^ cm^−2^	[85]
Pt/GO-SO_3_^−^/PPy ^(l)^	4-carboxyphenyl		c = 0.2 × 12 mM s = 0.56 μA mM^−1^ cm^−2^ In the presence of ascorbic acid (AA) and uric acid (UA)	[86]

^(a)^ CNT: carbon nanotube, GC: glassy carbon, GO: graphene oxide, HOPG: highly oriented pyrolytic graphite. ^(b)^ Fc: ferrocene, ^(c)^ 4-phenylacetic group + precursor film (PF) composed of one layer of poly(styrenesulfonate) (PSS) sandwiched between two layers of poly(dimethyldiallylammonium) (PDDA); GOx is cast on this layer-by-layer assembly and finally Nafion™ is deposited. ^(d)^ By reduction of the attached 4-nitrophenyl groups. ^(e)^ Fc-CH_2_-CH_2_-COOH attached to an amino function of the grafted aryl group. ^(f)^ Oxygen is used to reoxidize reduced GOx (GOxH_2_). ^(g)^ Interdigitated array of 1:1 aspect ratio carbon nanoelectrodes. ^(h)^ A crosslinked chitosan–glutaraldehyde–GOx gel is deposited on the Pt electrode. ^(i)^ Graphene is made porous by grafting diazonium salts, and GOx bonded to pyrenebutyric acid is adsorbed in the pores of HOPG. ^(j)^ Assembled by simultaneous reduction of two diazonium salts; 20-Å-long molecular wire. ^(k)^ Thionine diazonium cation is covalently attached onto the glassy carbon electrode via graphene nanosheets. ^(l)^ Nanocomposite layers are deposited onto platinum electrodes through the electrochemical polymerization of pyrrole monomer in the presence of reduced graphene oxide bearing phenylsulfonyl groups and further modified with carboxyphenyl groups via electrochemical reduction of 4-carboxybenzenediazonium.

**Table 3:** Detection of toxins. SWCNT—single-walled carbon nanotube.

Surface	Diazonium Salt + Attached Recognizing Group	Analyte	Detectable Label and Detection Limit	Reference
Attachment of toxins to surfaces modified by diazonium chemistry				
SPE ^(a)^	4-carboxybenzenediazonium + hexaethyleneglycol-modified 21-mer oligonucleotide	Aflatoxin M1 in milk 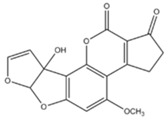	Direct detection by EIS ^(b)^ and CV ^(c)^c = 20–1000 ng/kg.	[97]
SPE	4-carboxybenzenediazonium+ anti-okadaic acid monoclonal antibody (anti-OA-MAb)	Okadaic acid 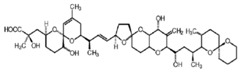	Direct detection by EIS	[98]
Graphene-modified SPE			Direct detection by SWV ^(d)^	[99]
SPE	4-carboxybenzenediazonium+ streptavidine + biotin-anti-TGFβ1	TGFβ1 protein	CV of viologen-modified SWCNT	[100]
SPR chip with Cu/graphene layer	3,5-bis-fluoro 4-carboxybenzenediazonium	HT-2 toxin 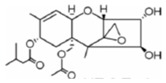	Label-free, surface plasmon resonance	[26]
Toxins modified with an aryl diazonium group				
Boron-doped diamond electrochemical microcell	Anti-ochratoxin polyclonal antibodies modified by a diazonium salt after coupling with 4-carboxymethylaniline	Ochratoxin A 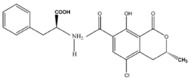		[101]

^(a)^ Carbon screen-printed electrode, ^(b)^ electrochemical impedance spectroscopy, ^(c)^ cyclic voltammetry, ^(d)^ square wave voltammetry.

**Table 4:** Detection of proteins. IgG—immunoglobulin G.

**Surface**	**Diazonium Salt + Attached Recognizing Group**	**Analyte**	**Detectable Label and Detection Limit**	**Reference**
Proteins modified with an aryl diazonium group				
SPE array	Immunoglobulin modified by a diazonium salt after coupling with 4-carboxymethylaniline	Anti-rabbit IgG antibodies	Peroxidase-labeled anti-rabbit or anti-human IgG antibodies. LOD: 50 fmol ^(a)^	[103]
SPE array	Anti-human IgG	Human IgG	Horseradish peroxidase (HRP)-modified secondary antibody. Detection limit: 60 nm human IgG	[104,105,106] ^(b)^
SPE array	Rabbit IgG modified by a diazonium salt after coupling with 4-carboxymethylaniline	Rheumatoid factor (RF)	Horseradish peroxidase (HRP)-modified secondary antibody. Detection range: 5.3–485 IU∙mL^−1^	
SPE array	HRP modified by a diazonium salt after coupling with 4-carboxymethylaniline	HRP		
Gold NPs ^(c)^ on SPE	Human prostate-specific antigen (PSA) modified by a diazonium salt after coupling with 4-carboxymethylaniline	Prostate-specific antigen (PSA) monoclonal antibody labeled with biotin	Horseradish peroxidase-labeled streptavidin Detection range of 5–80 ng/mL	[107]
(SPRi) ^(e)^ gold chip	Anti-ovalbumin IgG modified by a diazonium salt after coupling with 4-carboxymethylaniline	Ovalbumin	Direct reflectivity change. LOD: 100 ng/mL (2 nM).	[108] ^(d)^
GC, Au	Biotinylated anti-TNF-antibody (Tumor Necrosis Factor) modified by a diazonium salt after coupling with 4-carboxyaniline	Anti-TNF-antibody	Avidin modified gold NPs or avidin-HRP	[109] ^(e)^
Au	Carboxybetaine aryldiazonium derivative + lectin	Prostate-specific antigen (PSA)	EIS detection of anti-PSA antibody-modified Au nanoshells with a magnetic core	[110] ^(f)^
Attachment of proteins to surfaces modified by diazonium chemistry				
	**Modified Surface**	**Analyte**	**Detectable Label and Detection Limit**	
SPRi gold chip	Gold surface modified	Protein A	Direct reflectivity change upon coupling the protein to the attached carboxylic group	[111] ^(g)^
CNT	CNT modified by reaction of 4-carboxymethylbenzenediazonium + attachment of mouse olfactory receptor proteins	Various odoriferant organic compounds in vapor phase	Detection of odors: e-nose For example, 7 ppb of toluene	[112] ^(h)^
Graphene-modified GC	Graphene surface modified by reaction of 4-aminobenzenediazonium + coupling with A-Fetoprotein antibody	A-Fetoprein	HRP-labeled anti-AFP antibody LOD: 0.03 ng mL^−1^	[113] ^(i)^
SPE	Gold surface modified by reaction of 4-carboxybenzenediazonium	Lysozyme	Biotinylated antibody avidin–alkaline phosphatase LOD: 4.3 fM	[114] ^(j)^
Gold	Gold NPs modified with 4-mercaptoaniline, attached to gold surface by diazonium coupling	Five-amino-acid polypeptide with a biotin group	Peroxidase-labeled streptavidin	[115]
Nanocomposite: gold NPs loaded on reduced graphene oxide	Gold NPs modified with 4-carbxyphenyl and 4-aminophenylphosphorylcholine + coupling with anti-TNF-α capture antibody	Cytokine tumor necrosis factor-alpha (TNF-α)	Coupling anti-TNF-α detection antibody (Ab2) attached to graphene oxides modified with ferrocenyl groups. Electrochemical detection of ferrocene. LOD: 0.1 pg∙mL^−1^	[116] ^(k)^
Indium tin oxide (ITO)	Gold surface modified by reaction of the diazonium salt of 4-aminobutyric acid + peptidic coupling of antibody (Ab1)	Tumor necrosis factor	HRP-conjugated detection antibody (Ab2) LOD: 10 pg/mL	[117] ^(l)^
SPRi biochip	4-Carboxybenzenediazonium + anti-ovalbumin antibody	Ovalbumine	Direct detection by SPR	[118]
SPE	SPE surface modified by reaction of 4-methoxybenzenediazonium + oxidation of the grafted methoxy group+ attachment of anti-growth hormone antibodies	Growth hormone	EIS detection of 100 pg/mL growth hormone in undiluted whole blood LOD: 5 pg∙mL^−1^	[119] ^(m)^
Al–Mo nanoparticle membrane on Si	4-formylbenzenediazonium	Monoclonal antibodies specific for bovine herpes virus 1 (BHV-1)	Bovine herpes virus 1 Measurement of the resonance frequency shift of the membrane	[120]
Biomembrane-like films				
GC	Gold NPs capped with myoglobin on 4-carboxaldehyde diazonium salt *+* docecyl thiol	Myoglobin	Electrochemical detection of H_2_O_2_ 0.3 μM	[121]

^(a)^ LOD: Limit of Detection. Sequential modification of the electrodes is possible without interference; ^(b)^ the binding of non-RF human antibodies was found to be negligible; ^(c)^ NPs: nanoparticles; ^(c)^ SPRi: surface plasmon resonance imaging surface; ^(d)^ the integrity of the immobilized carboxymethylaniline-modified antibodies; the detection of ovalbumin by immobilized IgG against ovalbumin was evaluated; ^(e)^ response time 5 s, selective detection, selectivity of a given cytokine; ^(f)^ LOD: 34 fg∙mL^−1^and 108% recovery when spiking in a female serum; ^(g)^ non-specific absorption of the rabbit IgG remained negligible on the inter-spot areas; ^(h)^ remained active with stable normalized responses for ~5 days when stored in a humid environment; device responses to odorants show broad agreement with heterologous technique; ^(i)^ relative standard deviation was 4.5%, selectivity vs. bovine serum albumin and carcinoembryonic antigen, stability: 10 days under proper conditions; ^(j)^ detection range: 5 fM to 5 nM, selectivity vs. bovine serum albumin, cytochrome c, recovery 95–102% in wine samples; ^(k)^ selectivity vs. bovine serum albumin, prostate-specific antigen, cancer antigen-125, and mouse IgG, stability 30 days under proper conditions; ^(l)^ selectivity vs. hemoglobin, human serum albumin, comparison with ELISA test; ^(m)^ relative standard deviation less than 6% in whole-blood and plasma samples, mean recoveries ranging from 94% ± 3% to 103% ± 2%.

**Table 5:** Detection of DNA

Surface	Diazonium Salt + Attached Recognizing Group	Analyte	Detectable Label and Detection Limit	Reference
Modification of the Surface				
Carbon nanofibers	4-Nitrobenzenediazonium and reduction of 4-nitrophenyl to 4-aminophenyl groups after electrografting + reaction of a maleimide linker ^(a)^	Thiol-terminated DNA attaches to the linker	Fluorescently labeled, perfect complement to the grafted oligonucleotides	[123]
Vertically aligned diamond nano-wires	4-Nitrobenzenediazonium and reduction of 4-nitrophenyl to 4-aminophenyl groups after electrografting + maleimide linker ^(a)^ + thiol-terminated DNA, 23-mer cancer marker cytokeratin	The complementary DNA sequence	The complementary sequence detected by Differential Pulsed Voltammetry (DPV) LOD ~2 pM	[124] ^(b)^
Array of gold electrodes	4-Carboxybenzenediazonium and a bis-diazonium salt with a COOH terminal group + amino-terminated DNA	Human papillomavirus sequences terminated by tetramethylbenzidine	Electrochemical detection of tetramethylbenzidine	[127]
Vertically aligned carbon nanotubes	Diazonium salt of an aminophenyl group substituted _+_ β-cyclodextrin (β-CD)	DNA probe substituted on one end by a dabcyl group and a CdS nanoparticle at the other end	In the presence of the complementary sequence, the probe could be captured by the β-CD-modified CNT electrode LOD by DPV: 5.0 × 10^−13^ M	[128] ^(c)^
ITO, gold, GC	4-Carboxybenzenediazonium + attachment of avidin	Biotinylated DNA from influenza virus (type A)	Avidin–biotin recognition. Detection through the CV of ferro/ferricyanide LOD: 8.51 × 10^−14^ M	[122]
SPE	4-Nitrobenzenediazonium and reduction of 4-nitrophenyl to 4-aminophenyl groups after electrografting	Amine-modified (polyA)25 DNA probe.	Reaction between the diazonium group and NH_2_-DNA and recognition of the hybridization by EIS and DPV LOD: 4.65 nm	[129] ^(d)^
GC	4-Carboxybenzenediazonium and a naphthoquinone ^(e)^ to give a mixed layer + DNA probe attached to the surface	Fluorescent complementary DNA strand	Hybridization was detected by fluorescence and Alternative Current (AC) voltammetry Detection limit ca. 10 pM	[125]
Au	Mixed layer obtained from the diazonium salts bearing (i) a sulfobetaine group, (ii) a phenylmaleimido group + DNA probe attached to the surface	New Delhi metallo-β-lactamase (NDM)-Coding Gene	CV and chronoamperometric detection of the charges on the phosphate groups of DNA Detection limit: 54 pM	[130] ^(f)^
	The diazonium salts of 4-aminophenylacetic acid + covalent immobilization of streptavidin and incubation of a biotinylated DNA capture probe	Biotinylated DNA target sequence associated with the human papillomavirus	HRP-DNA probe + electrochemical detection of tetramethyl benzidine Detection limit: 0.50 nM	[126] ^(g)^
ITO	1-Naphthalenesulfonate diazonium salt	MicroRNA	Discrimination ability over single-mismatch, high sensitivity in the aM range thanks to the use of isothermal amplification strategy Detection limit: 25 aM	[131] ^(h)^
SPE	4-Carboxybenzenediazonium + peptidic attachment of a DNA probe		Determination of DNA damage by various reagents using EIS	[132] ^(i)^

^(a)^ Sulfosuccinimidyl-4-(*N*-maleimidomethyl) cyclohexane-1-carboxylate;^(b)^ concentration range: from 2 to 10 × 10^−12^ M, no degradation over 30 cycles of DNA hybridization/denaturation; ^(c)^ linear concentration range 1.0 × 10^−7^ to 1.0 × 10^−12^ M; ^(d)^ reproducibility: 7%, stability: retained 60% of its initial response after one month; ^(e)^ 5-hydroxy-1,4-naphthoquinone (juglone); ^(f)^ stability: >5 days; ^(g)^ linear range: 0–20 nM; ^(h)^ selectivity vs. concomitant miRNA, family members with sequence homology, recovery: 100% in 10% diluted human serum samples; ^(i)^ retained about 83% of its initial response after three weeks under proper conditions.

## References

[1] Allongue P., Delamar M., Desbat B., Fagebaume O., Hitmi R., Pinson J., Savéant J.-M. (1997). Covalent Modification of Carbon Surfaces by Aryl Radicals Generated from the Electrochemical Reduction of Diazonium Salts. J. Am. Chem. Soc..

[2] Berisha A., Chehimi M.M., Pinson J., Podvorica F.I. (2016). Electrode surface modification using diazonium salts. Electroanal. Chem..

[3] Chehimi M.M., Aryl Diazonium Salts (2012). New Coupling Agents in Polymer and Surface Science.

[4] Jiang C., Moraes Silva S., Fan S., Wu Y., Alam M.T., Liu G., Gooding J.J. (2017). Aryldiazonium salt derived mixed organic layers: From surface chemistry to their applications. J. Electroanal. Chem..

[5] Assresahegn B.D., Brousse T., Belanger D. (2015). Advances on the use of diazonium chemistry for functionalization of materials used in energy storage systems. Carbon.

[6] Delaporte N., Belanger R.L., Lajoie G., Trudeau M., Zaghib K. (2019). Multi-carbonyl molecules immobilized on high surface area carbon by diazonium chemistry for energy storage applications. Electrochim. Acta.

[7] Bondarev A.A., Naumov E.V., Kassanova A.Z., Krasnokutskaya E.A., Stankevich K.S., Filimonov V.D. (2019). First Study of the Thermal and Storage Stability of Arenediazonium Triflates Comparing to 4-Nitrobenzenediazonium Tosylate and Tetrafluoroborate by Calorimetric Methods. Org. Process Res. Dev..

[8] Pazo-Llorente R., Bravo-Diaz C., Gonzalez-Romero E. (2004). pH Effects on Ethanolysis of Some Arenediazonium Ions: Evidence for Homolytic Dediazoniation Proceeding through Formation of Transient Diazo Ethers. Eur. J. Org. Chem..

[9] Pazo-Llorente R., Maskill H., Bravo-Diaz C., Gonzalez-Romero E. (2006). Dediazoniation of 4-Nitrobenzenediazonium Ions in Acidic MeOH/H_2_O Mixtures: Role of Acidity and MeOH Concentration on the Formation of Transient Diazo Ethers that Initiate Homolytic Dediazoniation. Eur. J. Org. Chem..

[10] Berisha A., Combellas C., Kanoufi F., Decorse P., Oturan N., Médard J., Seydou M., Maurel M., Pinson J. (2017). Some Theoretical and Experimental Insights on the Mechanistic Routes Leading to the Spontaneous Grafting of Gold Surfaces by Diazonium Salts. Langmuir.

[11] Bui-Thi-Tuyet V., Cannizzo C., Legros C., Andrieux M., Chaussé A. (2019). Modification of fluorine-doped tin oxide surface: Optimization of the electrochemical grafting of diazonium salt. Surf. Interfaces.

[12] Griffete N., Ahmad R., Benmehdi H., Lamouri A., Decorse P., Mangeney C. (2013). Elaboration of hybrid silica particles using a diazonium salt chemistry approach. Colloids Surf. A.

[13] Schuurman J.C., McNeill A.R., Martinez-Gazoni R.F., Scott J.I., Reeves R.J., Allen M.W., Downard A.J. (2019). The effect of covalently bonded aryl layers on the band bending and electron density of SnO_2_ surfaces probed by synchrotron X-ray photoelectron spectroscopy. Phys. Chem. Chem. Phys..

[14] Gam-Derouich S., Pinson J., Decorse P., Luo Y., Herbaut R., Royon L., Mangeney C. (2018). Diazonium salt chemistry for the design of nano-textured anti-icing surfaces. Chem. Commun..

[15] Gam-Derouich S., Pinson J., Lamouri A., Decorse P., Bellynck S., Herbaut R., Royon L., Mangeney C. (2018). Micro-patterned anti-icing coatings with dual hydrophobic/hydrophilic properties. J. Mater. Chem. A.

[16] Joselevich M., Williams F.J. (2008). Synthesis and Characterization of Diazonium Functionalized Nanoparticles for Deposition on Metal Surfaces. Langmuir.

[17] Gorska B., Ratajczak P., Béguin F. (2019). Faradaic processes on quinone-grafted carbons in protic ionic liquid electrolyte. Electrochim. Acta.

[18] Hossain M.Z., Shimizu N. (2017). In Situ Functionalization of Graphene with Reactive End Group through Amine Diazotization. J. Phys. Chem. C.

[19] Thaçi B.S., Gashi S.T., Podvorica F.I. (2018). Preparation of heterogeneous reverse osmosis membranes undergoing modification process. Desalin. Water Treat..

[20] Zeb G., Viel P., Palacin S., Le X.T. (2015). On the chemical grafting of titanium nitride by diazonium chemistry. RSC Adv..

[21] Bouriga M., Chehimi M.M., Combellas C., Decorse P., Kanoufi F., Deronzier A., Pinson J. (2013). Sensitized Photografting of Diazonium Salts by Visible Light. Chem. Mater..

[22] Busson M., Berisha A., Combellas C., Kanoufi F., Pinson J. (2011). Photochemical grafting of diazonium salts on metals. Chem. Commun..

[23] Nguyen V.Q., Ai Y., Martin P., Lacroix J.-C. (2017). Plasmon-Induced Nanolocalized Reduction of Diazonium Salts. ACS Omega.

[24] Tijunelyte I., Kherbouche I., Gam-Derouich S., Nguyen M., Lidgi-Guigui N., Lamy de la Chapelle M., Lamouri A., Lévi G., Aubard J., Chevillot-Biraud A. (2018). Multi-functionalization of lithographically designed gold nanodisks by plasmon-mediated reduction of aryl diazonium salts. Nanoscale Horiz..

[25] Nguyen M., Kherbouche I., Gam-Derouich S., Ragheb I., Lau-Truong S., Lamouri A., Lévi G., Aubard J., Decorse P., Felidj N. (2017). Regioselective surface functionalization of lithographically designed gold nanorods by plasmon-mediated reduction of aryl diazonium salts. Chem. Commun..

[26] Wu F., Thomas P.A., Kravets V.G., Arola H.O., Soikkeli M., Iljin K., Kim G., Kim M., Shin H.S., Andreeva D.V. (2019). Layered material platform for surface plasmon resonance biosensing. Sci. Rep..

[27] Chehimi M.M., Pinson J. (2013). Applied Surface Chemistry of Nanomaterials.

[28] Unwin P.R., Guell A.G., Zhang G. (2016). Nanoscale Electrochemistry of sp2 Carbon Materials: From Graphite and Graphene to Carbon Nanotubes. Acc. Chem. Res..

[29] James D.K., Tour J.M. (2013). Graphene: Powder, Flakes, Ribbons, and Sheets. Acc. Chem. Res..

[30] Schirowski M., Hauke F., Hirsch A. (2019). Controlling the Degree of Functionalization: In-Depth Quantification and Side-Product Analysis of Diazonium Chemistry on SWCNTs. Chem. Eur. J..

[31] Romero-Ben E., Cid J.J., Assali M., Fernández-García E., Wellinger R.E., Khiar N. (2019). Surface modulation of single-walled carbon nanotubes for selective bacterial cell agglutination. Int. J. Nanomed..

[32] Clément P., Trinchera P., Cervantes-Salguero K., Ye Q., Jones C.R., Palma M. (2019). A One-Step Chemical Strategy for the Formation of Carbon Nanotube Junctions in Aqueous Solution: Reaction of DNA Wrapped Carbon Nanotubes with Diazonium Salts. ChemPlusChem.

[33] Ejigu A., Kinloch I.A., Dryfe R.A.W. (2017). Single Stage Simultaneous Electrochemical Exfoliation and Functionalization of Graphene. ACS Appl. Mater. Interfaces.

[34] Ossonon B.D., Bélanger D. (2017). Synthesis and characterization of sulfophenyl-functionalized reduced graphene oxide sheets. RSC Adv..

[35] Kirkman P.M., Güell A.G., Cuharuc A.S., Unwin P.R. (2014). Spatial and Temporal Control of the Diazonium Modification of sp2 Carbon Surfaces. J. Am. Chem. Soc..

[36] Bellunato A., Schneider G.F. (2018). Electrophilic radical coupling at the edge of Graphene. Nanoscale.

[37] Bernal M.M., Di Pierro A., Novara C., Giorgis F., Mortazavi B., Saracco G., Fina A. (2018). Edge-Grafted Molecular Junctions between Graphene Nanoplatelets: Applied Chemistry to Enhance Heat Transfer in Nanomaterials. Adv. Funct. Mater..

[38] van Nguyen Q., Martin P., Frath D., Della Rocca M.L., Lafolet F., Barraud C., Lafarge P., Mukundan V., James D., McCreery R.L. (2017). Control of rectification in molecular junctions: Contact effects and molecular signature. J. Am. Chem. Soc..

[39] McCreery R.L., Bergren A.J. (2009). Progress with Molecular Electronic Junctions: Meeting Experimental Challenges in Design and Fabrication. Adv. Mater..

[40] Supur M., Van Dyck C., Bergren A.J., McCreery R.L. (2018). Bottom-up, Robust Graphene Ribbon Electronics in All-Carbon Molecular Junctions. ACS Appl. Mater. Interfaces.

[41] Liu Z., Gan F., Dong N., Zhang B., Wang J., Chen Y. (2019). Fabrication and nonlinear optical characterization of fluorinated zinc phthalocyanine covalently modified black phosphorus/PMMA films using the nanosecond Z-scan technique. J. Mater. Chem. C.

[42] Li D.O., Gilliam M.S., Chu X.S., Yousaf A., Guo Y., Green A.A., Wang Q.H. (2019). Covalent chemical functionalization of semiconducting layered chalcogenide nanosheets. Mol. Syst. Des. Eng..

[43] Wang H., Zhang J., Wu Y., Huang H., Jiang Q. (2018). Chemically functionalized two-dimensional titanium carbide MXene by in situ grafting-intercalating with diazonium ions to enhance supercapacitive performance. J. Phys. Chem. Solids.

[44] Kainz Q.M., Reiser M. (2014). Polymer- and Dendrimer-Coated Magnetic Nanoparticles as Versatile Supports for Catalysts, Scavengers, and Reagents. Acc. Chem. Res..

[45] Griffete N., Herbst F., Pinson J., Ammar S., Mangeney C. (2011). Preparation of Water-Soluble Magnetic Nanocrystals Using Aryl Diazonium Salt Chemistry. J. Am. Chem. Soc..

[46] Charlotte Boitard C., Lamouri M.C., Griffete N. (2019). Whole Protein Imprinting over Magnetic Nanoparticles Using Photo-Polymerization. ACS Appl. Polym. Mater..

[47] Sanders S., Golden T.D. (2019). Functionalization of Cerium Oxide Nanoparticles to Influence Hydrophobic Properties. Langmuir.

[48] Mousli F., Chaouchi A., Hocine S., Lamouri A., Rei Vilar M., Kadri A., Chehimi M.M. (2019). Diazonium-modified TiO_2_/polyaniline core/shell nanoparticles. Structural characterization, interfacial aspects and photocatalytic performances. Appl. Surf. Sci..

[49] Jasmin J.-P., Cannizzo C., Dumas E., Chaussé A. (2014). Fabrication and characterization of all-covalent nanocomposite functionalized screen-printed voltammetric sensors. Electrochim. Acta.

[50] Mooste M., Kibena-Põldsepp E., Diby Ossonon B., Bélanger D., Tammeveski K. (2018). Oxygen reduction on graphene sheets functionalised by anthraquinone diazonium compound during electrochemical exfoliation of graphite. Electrochim. Acta.

[51] Laurentius L., Stoyanov S.R., Gusarov S., Kovalenko A., Du R., Lopinski G.P., McDermott M.T. (2011). Diazonium-derived aryl films on gold nanoparticles: Evidence for a carbon gold covalent bond. ACS Nano.

[52] Sampathkumar K., Diez-Cabanes V., Kovaricek P., del Corro E., Bouša M., Hošek J., Kalbac M., Frank O. (2019). On the Suitability of Raman Spectroscopy to Monitor the Degree of Graphene Functionalization by Diazonium Salts. J. Phys. Chem. C.

[53] Berger F.J., Luttgens J., Nowack T., Kutsch T., Lindenthal S., Kistner L., Muller C.C., Bongartz L.M., Lumsargis V.A., Zakharko Y. (2019). Brightening of Long, Polymer—Wrapped Carbon Nanotubes by sp^3^ Functionalization in Organic Solvents. ACS Nano.

[54] Wepasnick K.A., Smith B.A., Bitter J.L., Fairbrother D.H. (2010). Chemical and structural characterization of carbon nanotube surfaces. Anal. Bioanal. Chem..

[55] Van Dyck C., Bergren A.J., Mukundan V., Fereiro J.A., DiLabio G.A. (2019). Extent of conjugation in diazonium-derived layers in molecular junction devices determined by experiment and modelling. Phys. Chem. Chem. Phys..

[56] Descroix S., Hallais G., Corinne Lagrost C., Pinson J. (2013). Regular poly (para-phenylene) films bound to gold surfaces through the electrochemical reduction of diazonium salts followed by electropolymerization in an ionic liquid. Electrochim. Acta.

[57] Greenwood J., Phan T.H., Fujita Y., Li Z., Ivasenko O., Vanderlinden W., Van Gorp H., Frederickx W., Lu G., Tahara K. (2015). Covalent Modification of Graphene and Graphite Using Diazonium Chemistry: Tunable Grafting and Nanomanipulation. ACS Nano.

[58] Anariba F., DuVall S.H., McCreery R.L. (2003). Mono- and Multilayer Formation by Diazonium Reduction on Carbon Surfaces Monitored with Atomic Force Microscopy “Scratching”. Anal. Chem..

[59] Ceccato M., Bousquet A., Hinge M., Pedersen S.U., Daasbjerg K. (2011). Using a Mediating Effect in the Electroreduction of Aryldiazonium Salts To Prepare Conducting Organic Films of High Thickness. Chem. Mater..

[60] Combellas C., Kanoufi F., Pinson J., Podvorica F.I. (2008). Sterically Hindered Diazonium Salts for the Grafting of a Monolayer on Metals. J. Am. Chem. Soc..

[61] Leroux Y.R., Fei H., Noël J.-M., Roux C., Hapiot P. (2010). Efficient covalent modification of a carbon surface: Use of a silyl protecting group to form an active monolayer. J. Am. Chem. Soc..

[62] Pichereau L., López I., Cesbron M., Dabos-Seignon S., Gautier C., Breton T. (2019). Controlled diazonium electrografting driven by overpotential reduction: A general strategy to prepare ultrathin layers. Chem. Commun..

[63] Xia Z., Leonardi F., Gobbi M., Liu Y., Bellani V., Liscio A., Kovtun A., Li R., Feng X., Orgiu E. (2016). Electrochemical Functionalization of Graphene at the Nanoscale with Self-Assembling Diazonium Salts. ACS Nano.

[64] Dincer C., Bruch R., Costa-Rama E., Fernández-Abedul M.T., Merkoçi A., Manz A., Urban G.A., Güder F. (2019). Disposable Sensors in Diagnostics, Food, and Environmental Monitoring. Adv. Mater..

[65] Gooding J.J. (2008). Advances in interfacial design sensors: Aryl diazonium salts for electrochemical biosensors and for modifying carbon and metal electrodes. Electroanalysis.

[66] Mahouche-Chergui S., Gam-Derouich S., Mangeney C., Chehimi M.M. (2011). Aryl diazonium salts: A new class of coupling agents for bonding polymers, biomacromolecules and nanoparticles to surfaces. Chem. Soc. Rev..

[67] Zhou Y., Chiu C.-W., Liang H. (2012). Interfacial Structures and Properties of Organic Materials for Biosensors: An Overview. Sensors.

[68] Abdellaoui S., Corgier B.C., Mandon C.A., Doumèche B., Marquette C.A., Blum L.J. (2013). Biomolecules Immobilization Using the Aryl Diazonium Electrografting. Electroanalysis.

[69] Cao C., Zhang Y., Jiang C., Qi M., Liu G. (2017). Advances on Aryldiazonium Salt Chemistry Based Interfacial Fabrication for Sensing Applications. ACS Appl. Mater. Interfaces.

[70] Yáñez-Sedeño P., Campuzano S., Pingarrón J.M. (2018). Integrated Affinity Biosensing Platforms on Screen-Printed Electrodes Electrografted with Diazonium Salts. Sensors.

[71] Yates N.D.J., Fascione M.A., Parkin A. (2018). Methodologies for “Wiring” Redox Proteins/Enzymes to Electrode Surfaces. Chem. A Eur. J..

[72] Yáñez-Sedeño P., González-Cortés A., Campuzano S., Pingarrón J.M. (2019). Copper(I)-Catalyzed Click Chemistry as a Tool for the Functionalization of Nanomaterials and the Preparation of Electrochemical (Bio)Sensors. Sensors.

[73] Bourdillon C., Delamar M., Demaille C., Hitmi R., Moiroux J., Pinson J. (1992). Immobilization of glucose oxidase on a carbon surface derivatized by electrochemical reduction of diazonium salts. J. Electroanal. Chem..

[74] Yang X., Hall S.B., Tan S.N. (2003). Electrochemical Reduction of a Conjugated Cinnamic Acid Diazonium Salt as an Immobilization Matrix for Glucose Biosensor. Electroanalysis.

[75] Liu G., Paddon-Row M.N., Gooding J.J. (2007). A molecular wire modified glassy carbon electrode for achieving direct electron transfer to native glucose oxidase. Electrochem. Commun..

[76] Qi M., Zhang Y., Cao C., Luc Y., Liu G. (2016). Increased sensitivity of extracellular glucose monitoring based on AuNP decorated GO nanocomposites. RSC Adv..

[77] Ferreyra N., Coche-Guérente L., Labbé P. (2004). Construction of layer-by-layer self-assemblies of glucose oxidase and cationic polyelectrolyte onto glassy carbon electrodes and electrochemical study of the redox-mediated enzymatic activity. Electrochim. Acta.

[78] Zhao K., Zhuang S., Chang Z., Songm H., Dai L., He P., Fanga Y. (2007). Amperometric Glucose Biosensor Based on Platinum Nanoparticles Combined Aligned Carbon Nanotubes Electrode. Electroanalysis.

[79] Le Goff A., Moggia F., Debou N., Jegou P., Artero V., Fontecave M., Jousselme B., Palacin S. (2010). Facile and tunable functionalization of carbon nanotube electrodes with ferrocene by covalent coupling and p-stacking interactions and their relevance to glucose bio-sensing. J. Electroanal. Chem..

[80] Nasri A., Shams E., Ahmadi M. (2013). A glucose biosensor based on direct attachment of in situ generated Nile blue diazonium cations to the electrode surface. J. Electroanal. Chem..

[81] Sharma D., Lim Y., Lee Y., Shin H. (2015). Glucose sensor based on redox-cycling between selectively modified and unmodified combs of carbon interdigitated array nanoelectrodes. Anal. Chim. Acta.

[82] Raicopol M.D., Andronescu C., Atasiei R., Hanganu A., Vasile E., Brezoiu A.M., Pilan L. (2016). Organic layers via aryl diazonium electrochemistry: Towards modifying platinum electrodes for interference free glucose biosensors. Electrochim. Acta.

[83] Li M., Cui L., Niu F., Ji X., Xu Y., Liu J. (2017). Efficient and Facile Fabrication of Glucose Biosensor Based on Electrochemically Etched Porous HOPG platform. Electroanalysis.

[84] Morita K., Hirayama N., Imura H., Yamaguchi A., Teramae N. (2011). Grafting of phenylboronic acid on a glassy carbon electrode and its application as a reagentless glucose sensor. J. Electroanal. Chem..

[85] Shervedani R.K., Amini A., Sadeghi N. (2016). Electrografting of thionine diazonium cationon to the graphene edges and decorating with Au nano-dendrites or glucoseoxidase: Characterization and electrocatalytic applications. Biosens. Bioelectron..

[86] Ott C., Raicopol M.D., Andronescu C., Vasile E., Hanganue A., Prunaf A., Pilan L. (2018). Functionalized polypyrrole/sulfonated graphene nanocomposites: Improved biosensing platforms through aryl diazonium electrochemistry. Synth. Met..

[87] Harper J.C., Polsky R., Dirk S.M., Wheeler D.R., Brozik S.M. (2007). Electroaddressable Selective Functionalization of Electrode Arrays: Catalytic NADH Detection Using Aryl Diazonium Modified Gold Electrodes. Electroanalysis.

[88] Nasri Z., Shams E., Ahmadi M. (2007). Direct Modification of a Glassy Carbon Electrode with ToluidineBlue Diazonium Salt: Application to NADH Determination and Biosensing of Ethanol. Electroanalysis.

[89] Venarussoa L.B., Tammeveski K., Maia G. (2011). Versatile charge transfer through anthraquinone films for electrochemical sensing applications. Electrochim. Acta.

[90] Revenga-Parra M., Gomez-Anquela C., Garcia-Mendiola T., Gonzalez E., Parientea F., Lorenzo E. (2012). Grafted Azure A modified electrodes as disposable β-nicotinamide adenine dinucleotide sensors. Anal. Chim. Acta.

[91] Kesavan S., Revin S.B., John S.A. (2014). Potentiodynamic formation of gold nanoparticles film on glassy carbon electrode using aminophenyl diazonium cations grafted gold nanoparticles: Determination of histamine H2 receptor antagonist. Electrochim. Acta.

[92] Alarfaj N.A., El-Tohamy M.F. (2017). A label-free electrochemical immunosensor based on gold nanoparticles and graphene oxide for the detection of tumor marker calcitonin. New J. Chem..

[93] William Richard W., Evrard D., Gros P. (2014). A Novel Electrochemical Sensor Based on a Mixed Diazonium/PEDOT Surface Functionalization for the Simultaneous Assay of Ascorbic and Uric Acids. Towards an Improvement in Amperometric Response Stability. Electroanalysis.

[94] Assaud L., Massonnet N., Evrard D., Vergnes H., Salvagnac L., Conédéra V., Noé L., Monthioux M., Gros P., Temple-Boyer P. (2016). A new route for the integration of a graphene/diazonium/PEDOT electrode towards antioxidant biomarker detection. J. Electroanal. Chem..

[95] Himori S., Nishitani S., Sakata T. (2019). Control of Potential Response to Small Biomolecules with Electrochemically Grafted Aryl-Based Monolayer in Field-Effect Transistor-Based Sensors. Langmuir.

[96] Rather J.A., Khudaish E.A., Kannan P. (2018). Graphene-amplified femtosensitive aptasensing of estradiol, an endocrine disruptor. Analyst.

[97] Istamboulié G., Paniel N., Zara L., Reguillo Granados L., Barthelmebs L., Noguer T. (2016). Development of an impedimetric aptasensor for the determination of aflatoxin M1 in milk. Talanta.

[98] Hayat A., Barthelmebs L., Marty J.-L. (2012). Electrochemical impedimetric immunosensor for the detection of okadaic acid in mussel sample. Sens. Actuators B.

[99] Eissa S., Zourob M. (2012). A graphene-based electrochemical competitive immunosensor for the sensitive detection of okadaic acid in shellfish. Nanoscale.

[100] Sánchez-Tirado E., Arellano L.M., González-Cortés A., Yáñez-Sedeño P., Langa F., Pingarrón J.M. (2017). Viologen-functionalized single-walled carbon nanotubes as carrier nanotags for electrochemical immunosensing. Application to TGF-β1 cytokine. Biosens. Bioelectron..

[101] Chrouda A., Sbartai A., Bessueille F., Renaud L., Maaref A., Jaffrezic-Renault N. (2015). Electrically addressable deposition of diazonium functionalized antibodies on boron-doped diamond microcells for the detection of ochratoxin A. Anal. Methods.

[102] Alonso-Lomillo M.A., Domínguez-Renedo O., Matos P., Arcos-Martín M.J. (2010). Disposable biosensors for determination of biogenic amines. Anal. Chim. Acta.

[103] Corgier B.P., Marquette C.A., Blum L.J. (2005). Diazonium-Protein Adducts for Graphite Electrode Microarrays Modification: Direct and Addressed Electrochemical Immobilization. J. Am. Chem. Soc..

[104] Corgier B.P., Laurent A., Perriat P., Blum L.J., Marquette C.A. (2007). A Versatile Method for Direct and Covalent Immobilization of DNA and Proteins on Biochips. Angew. Chem. Int. Ed..

[105] Corgier B.P., Li F., Blum L.J., Marquette C.A. (2007). Direct electrochemical addressing of immunoglobulins: Immuno-chip on screen-printed microarray. Biosens. Bioelectron..

[106] Marquette C.A., Bouteille F., Corgier B.P., Degiuli A., Blum L.J. (2009). Disposable screen-printed chemiluminescent biochips for the simultaneous determination of four point-of-care relevant proteins. Anal. Bioanal. Chem..

[107] Corgier B.P., Li F., Blum L.J., Marquette C.A. (2007). On-Chip Chemiluminescent Signal Enhancement Using Nanostructured Gold-Modified Carbon Microarrays. Langmuir.

[108] Corgier B.P., Bellon S., Anger-Leroy M., Blum L.J., Marquette C.A. (2009). Protein-Diazonium Adduct Direct Electrografting onto SPRi-Biochip. Langmuir.

[109] Polsky R., Harper J.C., Wheeler D.R., Dirk S.M., Arango D.C., Brozik S.M. (2008). Electrically addressable diazonium-functionalized antibodies for multianalyte electrochemical sensor applications. Biosens. Bioelectron..

[110] Bertok T., Lorencova L., Hroncekova S., Gajdosova V., Jane E., Hires M., Kasak P., Kaman O., Sokol R., Bella V. (2019). Advanced impedimetric biosensor configuration and assay protocol for glycoprofiling of a prostate oncomarker using Au nanoshells with a magnetic core. Biosens. Bioelectron..

[111] Mandon C.A., Blum L.J., Marquette C.A. (2009). Aryl Diazonium for Biomolecules Immobilization onto SPRi Chips. ChemPhysChem.

[112] Goldsmith B.R., Mitala J.J., Josue J., Castro A., Lerner M.B., Bayburt T.H., Khamis S.M., Jones R.A., Brand J.G., Sligar S.G. (2011). Biomimetic Chemical Sensors Using Nanoelectronic Readout of Olfactory Receptor Proteins. ACS Nano.

[113] Wang R., Xue C. (2013). A sensitive electrochemical immunosensor for alpha-fetoprotein based on covalently incorporating a biorecognition element onto a graphene modified electrode via diazonium chemistry. Anal. Methods.

[114] Ocaña C., Hayat A., Mishra R., Vasilescu A., del Vallea M., Marty J.-L. (2015). A novel electrochemical aptamer–antibody sandwich assay for lysozyme detection. Analyst.

[115] Adabo A.H., Zeggari R., Saïd N.M., Bazzi R., Elie-Caille C., Marquette C., Martini M., Tillement O., Perriat P., Chaix C. (2016). Enhanced chemiluminescence-based detection on gold substrate after electrografting of diazonium precursor-coated gold nanoparticles. J. Coll. Interface Sci..

[116] Qi M., Zhang Y., Cao C., Zhang M., Liu S., Liu G. (2016). Decoration of Reduced Graphene Oxide Nanosheets with Aryldiazonium Salts and Gold Nanoparticles toward a Label-Free Amperometric Immunosensor for Detecting Cytokine Tumor Necrosis Factor-α in Live Cells. Anal. Chem..

[117] Jiang C., Alam M.T., Moraes Silva S., Taufik S., Fan S., Gooding J.J. (2016). Unique Sensing Interface That Allows the Development of an Electrochemical Immunosensor for the Detection of Tumor Necrosis Factor α in Whole Blood. ACS Sens..

[118] Fioresi F., Rouleau A., Maximova K., Vieillard J., Boireau W., Elie Caille C., Soulignac C., Zeggarib R., Clamens T., Lesouhaitier O. (2019). Electrografting of diazonium salt for SPR application. Mater. Today Proc..

[119] Li N., Chow A.M., Ganesh H.V.S., Ratnam M., Brown I.R., Kerman K. (2019). Diazonium-Modified Screen-Printed Electrodes for Immunosensing Growth Hormone in Blood Samples. Biosensors.

[120] van den Hurk R., Baghelani M., Chen J., Daneshmand M., Evoy S. (2019). Al-Mo nanocomposite functionalization for membrane-based resonance detection of bovine Herpesvirus-1. Sens. Actuators A.

[121] Anjum S., Qi W., Gao W., Zhao J., Hanif S., Rehman A.-U., Xu G. (2015). Fabrication of biomembrane-like films on carbon electrodes using alkanethiol and diazonium salt and their application for direct electrochemistry of myoglobin. Biosens. Bioelectron..

[122] Chung D.-J., Oh S.-H., Komathi S., Gopalan A.I., Lee K.-P., Choi S.-H. (2012). One-step modification of various electrode surfaces using diazonium salt compounds and the application of this technology to electrochemical DNA (E-DNA) sensors. Electrochim. Acta.

[123] Baker S.E., Tse K.-Y., Hindin E., Nichols B.M., Clare T.L., Robert J., Hamers R.J. (2005). Covalent Functionalization for Biomolecular Recognition on Vertically Aligned Carbon Nanofibers. Chem. Mater..

[124] Nebel C.E., Yang N., Uetsuka H., Osawa E., Tokuda N., Williams O. (2009). Diamond nano-wires, a new approach towards next generation electrochemical gene sensor platforms. Diam. Relat. Mater..

[125] Hai L.V., Reisberg S., Chevillot-Biraud A., Noël V., Pham M.C., Piro B. (2014). Simultaneous Electroreduction of Different Diazonium Salts for Direct Electrochemical DNA Biosensor Development. Electrochim. Acta.

[126] Bartolome J.P., Echegoyen L., Fragoso A. (2015). Reactive Carbon Nano-Onion Modified Glassy Carbon Surfaces as DNA Sensors for Human Papillomavirus Oncogene Detection with Enhanced Sensitivity. Anal. Chem..

[127] Civit L., Fragoso A., O’Sullivan C.K. (2010). Thermal stability of diazonium derived and thiol-derived layers on gold for application in genosensors. Electrochem. Commun..

[128] Yang L., Xu Y., Wang X., Zhu J., Zhang R., He P., Fang Y. (2011). The application of cyclodextrin derivative functionalized aligned carbon nanotubes for electrochemically DNA sensing via host–guest recognition. Anal. Chim. Acta.

[129] Revenga-Parra M., Garcia-Mendiola T., Gonzalez-Costas J., Gonzalez-Romero E., Garcia Marin A., Pau J.L., Pariente F., Lorenzo E. (2014). Simple diazonium chemistry to develop specific gene sensing platforms. Anal. Chim. Acta.

[130] Kuo T.M., Shen M.Y., Huang S.Y., Li Y.K., Chuang M.C. (2016). Facile Fabrication of a Sensor with a Bifunctional Interface for Logic Analysis of the New Delhi Metallo-β-Lactamase (NDM)-Coding Gene. ACS Sens..

[131] Ge L., Wang W., Li F. (2017). Electro-Grafted Electrode with Graphene-Oxide-Like DNA Affinity for Ratiometric Homogeneous Electrochemical Biosensing of MicroRNA. Anal. Chem..

[132] Mousavisania S.Z., Raoof J.-B., Turner A.P.F., Ojani R., Mak W.C. (2018). Label-free DNA sensor based on diazonium immobilisation for detection of DNA damage in breast cancer 1 gene. Sens. Actuators B.

[133] Guselnikova O., Dvorankova B., Kakisheva K., Kalachyova Y., Postnikov P., Svorcik V., Lyutakov O. (2019). Rapid SERS-based recognition of cell secretome on the folic acid-functionalized gold gratings. Anal. Bioanal. Chem..

